# Supplementary material for: Topology comparison of Twitter diffusion networks effectively reveals misleading information
Source: Sci Rep. 2020 Jan 28;10:1372. doi: 10.1038/s41598-020-58166-5 (PMC6987152; doi:10.1038/s41598-020-58166-5)
Supplement: Supplementary file 1 — Supplementary Information. [file 41598_2020_58166_MOESM1_ESM.pdf]

Topology comparison of Twitter diffusion networks effectively reveals misleading information

Francesco Pierri, Carlo Piccardi, and Stefano Ceri

January 2, 2020

1 Mainstream and misleading news

1.1 Collecting mainstream news on Twitter

In order to gather mainstream news diffusion networks we followed the same approach as in [17]: we first used Twitter Streaming API, via `tweepy`[1] package in Python, to filter all tweets containing an URL matching the domains of top trusted sources specified in [12]. Among sources described in the research report, we only selected most reliable sources listed in Table 1 (the bias is derived according to [4]). In particular we specified domains URLs as *track* parameter to call Twitter Api, e.g. "wsj com OR nytimes com OR news yahoo com. OR ..." as suggested by Twitter Developers documentation[2]. The tweets collected in this way contained more than 300k unique URL; about 12k of them were associated with at least 50 tweets, further reduced to 6978 after handling censoring effects described in [7].

[1] `http://docs.tweepy.org/en/v3.5.0/`

[2] `https://developer.twitter.com/en/docs/tweets/filter-realtime/guides/basic-stream-parameters`

**Newspaper**	**Domain**	**Bias**
The Wall Street Journal	wsj.com	Right
The New York Times	nytimes.com	Left
The Washington Post	washingtonpost.com	Left
USA Today	usatoday.com	Centre
CNN	cnn.com	Centre
ABCNews	abcnews.go.com	Left
Bloomberg	bloomberg.com	Centre
Fox News	foxnews.com	Right
PBS	pbs.org	Centre
NPR	npr.org	Centre
CBS News	cbsnews.org	Left
NBC News	nbcnews.org	Left
The Economist	economist.com	Centre
MSNBC	msnbc.com	Left
The Guardian	theguardian.com	Left
The New Yorker	newyorker.com	Left
Politico	politico.com	Centre
Yahoo News	news.yahoo.com	Centre

Table 1: List of monitored mainstream news domains.

1.2 Misleading sources

In Table 2 we provide bias labels for misleading news outlets; we indicate fewer sources w.r.t to the original list provided by [16] (and available at `https://docs.google.com/spreadsheets/d/1S5eDzOUEByRcHSwSNmSqjQMpaKcKXmUzYT6YlRy3UOg/edit#gid=1882442466`), i.e. sources with at least one news article in our dataset. Bias labels are obtained resorting to ”allside.com” and ”mediabias-factcheck.com”, as in [4], and we indicate missing labels with ”-”.

**Outlet**	**Bias**
breitbart.com	Right
politicususa.com	Left
redstate.com	Right
infowars.com	Right
theblaze.com	Right
activistpost.com	Left
dcclothesline.com	Right
theonion.com	Satire
thefreethoughtproject.com	Left
wnd.com	Right
lewrockwell.com	Right
beforeitsnews.com	-
naturalnews.com	Right
twitchy.com	Right
govtslaves.info	-
21stcenturywire.com	Left
globalresearch.ca	Left
worldtruth.tv	-
anonews.co	-
disclose.tv	-
realfarmacy.com	-
burrardstreetjournal.com	-
gomerblog.com	Satire
huzlers.com	-
coasttocoastam.com	-
geoengineeringwatch.org	-
worldnewsdailyreport.com	-
clickhole.com	Satire
duffelblog.com	Satire
bipartisanreport.com	Left
nowtheendbegins.com	Right
veteranstoday.com	-

Table 2: List of misleading outlets collected in our dataset.

1.3 Composition of the dataset

We provide in Fig 1 and Fig 2 (respectively for *mainstream* and *misleading* news) barplots for the distribution of articles according to different sources and bias. Only 98.5% of misleading news networks is present as some sources do not have a bias label. We further provide in Table 3 a concise breakdown of the dataset of network cascades according to both class and bias labels.

Class	Bias	No. networks
Mainstream	Left	4573
Mainstream	Centre	1079
Mainstream	Right	1292
Misleading	Left	1052
Misleading	Satire	444
Misleading	Right	4194

Table 3: Breakdown of the dataset of networks in terms of class and bias labels.

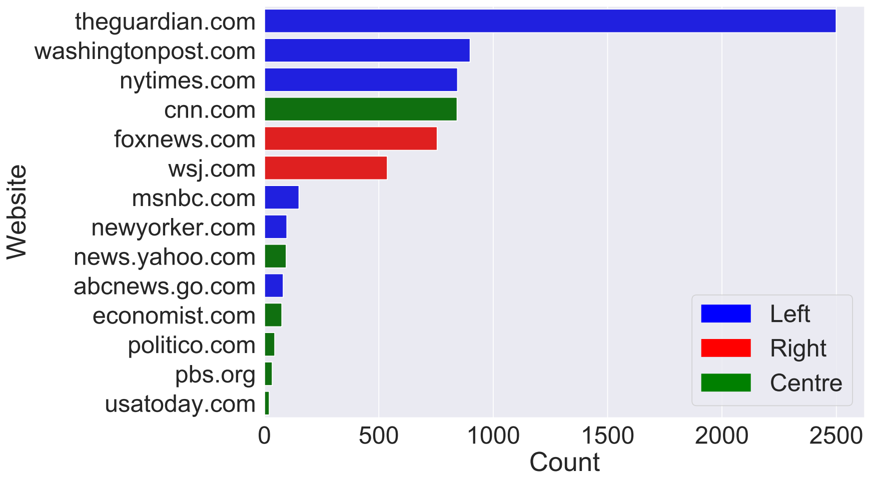

Figure 1: Distribution of the number of networks per mainstream source. Colors indicate the bias of the source as specified in the legend.

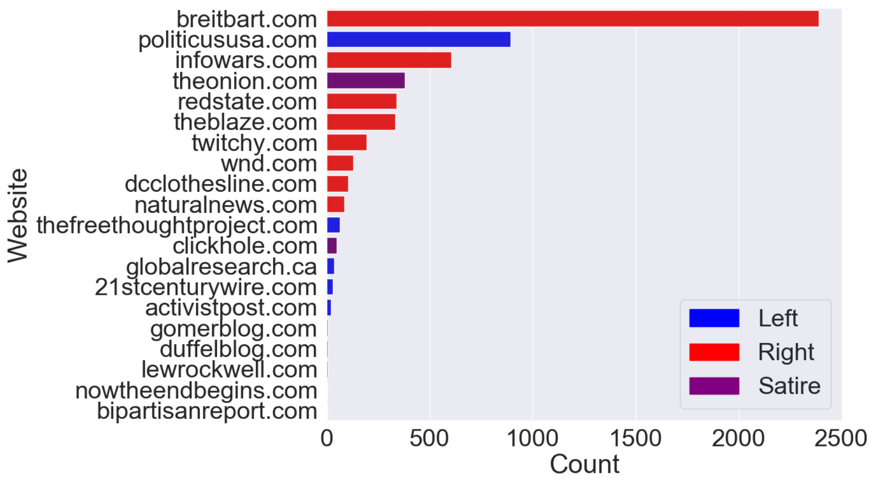

Figure 2: Distribution of the number of networks per misleading source. Colors indicate the bias of the source as specified in the legend.

2 Network Comparison Approaches

2.1 Global Network properties

We considered the following global network properties:

1. **Number of Strongly Connected Components (SCC)**: a Strongly Connected Component of a directed graph is a maximal (sub)graph where for each pair of vertices u, v there is a path in each direction ($u \to v$, $v \to u$).

2. **Size of the Largest Strongly Connected Component (LSCC)**: the number of nodes in the largest strongly connected component of a given graph.

3. **Number of Weakly Connected Components (WCC)**: a Weakly Connected Component of a directed graph is a maximal (sub)graph where for each pair of vertices (u, v) there is a path $u \leftrightarrow v$ ignoring edge directions.

4. **Size of the Largest Weakly Connected Component (LWCC)**: the number of nodes in the largest weakly connected component of a given graph.

5. **Diameter of the Largest Weakly Connected Component (DWCC)**: the largest distance (length of the shortest path) between two nodes in the largest weakly connected component of a graph.

6. **Average Clustering Coefficient (CC)**: the average of the local clustering coefficients of all nodes in a graph; the local clustering coefficient of a node quantifies how close its neighbours are to being a complete graph (or a clique). It is computed according to [15].

7. **Main K-core Number (KC)**: a K-core [3] of a graph is a maximal subgraph that contains nodes of internal degree k or more; the main K-core number is the highest value of k (in directed and weighted graphs the total degree is considered).

This is a non-exhaustive list of classical properties which can be used to describe a network [1, 13].

2.2 Centrality Measures

We computed the following centrality measures as provided in `networkx` package [8]: *Betweenness*, *Clustering*, *Closeness*, *Degree*, *Eigenvector*, *In-Degree*, *Katz*, *Load* (or *Newman's Betweenness*) and *Out-Degree*.
References and details on how these measures are computed are available in

`networkx` documentation[3]. In particular *Degree*, *In-Degree* and *Out-Degree* distributions are normalized, e.g. for each node the corresponding centrality value is divided by $N - 1$ (the maximum degree of a graph) where N is the number of nodes in the graph. For what concerns the similarity matrix, we computed Wasserstein Distance between empirical distributions using `scipy` Python package [9] and `numpy` [18] Python package to compute the empirical distribution function (with `no_bins`=100).

3 Analysis of Global Network Properties

3.1 Statistical Tests

In order to assess statistical differences between features distributions we used the non-parametric Kolmogorov Smirnov (KS) two-sample test, as provided by `scipy` Python package [9]. In tables S2 to S5 we report KS statistic and associated p-value for all features (plus network size) in all subsets; as usual we reject the null hypothesis (a given feature has the same distribution in the two domains) when we observe p-value ¡ $\alpha = 0.05$.

Kolmogorov Smirnov two-sample test for all features in $D_{[0,100)}$

**Feature**	KS statistic	KS p-value
Size	0.2716	3.0831e-104
SCC	0.2658	6.5331e-100
LSCC	0.1857	5.9470e-49
WCC	0.0987	3.7962e-14
LWCC	0.1483	2.1263e-31
DWCC	0.2687	4.4415e-102
CC	0.0654	1.8972e-06
KC	0.2189	7.1959e-68

Table 4: Kolmogorov-Smirnov statistic and p-value for all features in $D_{[0,100)}$.

Kolmogorov Smirnov two-sample test for all features in $D_{[100,1000)}$

**Feature**	KS statistic	KS p-value
Size	0.0535	7.2564e-04
SCC	0.0512	1.3914e-03
LSCC	0.4363	1.4851e-229
WCC	0.3519	1.8491e-149
LWCC	0.2698	4.7916e-88
DWCC	0.3059	4.3610e-113
CC	0.2750	1.7796e-91
KC	0.4055	2.1375e-198

Table 5: Kolmogorov-Smirnov statistic and p-value for all features in $D_{[100,1000)}$.

[3] `https://networkx.github.io/documentation/stable/reference/algorithms/centrality.html`

Kolmogorov Smirnov two-sample test for all features in $D_{[1000,+\infty)}$

**Feature**	KS statistic	KS p-value
Size	0.0606	8.1456e-01
SCC	0.0606	8.1456e-01
LSCC	0.5617	1.6478e-30
WCC	0.4854	6.8044e-23
LWCC	0.1908	6.7693e-04
DWCC	0.1351	3.6397e-02
CC	0.3997	1.1645e-15
KC	0.0916	3.1587e-01

Table 6: Kolmogorov-Smirnov statistic and p-value for all features in $D_{[1000,+\infty)}$.

Kolmogorov Smirnov two-sample test for all features in D_{all}

**Feature**	KS statistic	KS p-value
Size	0.1862	5.2890e-96
SCC	0.1833	4.3347e-93
LSCC	0.3371	9.4902e-314
WCC	0.1202	3.1607e-40
LWCC	0.2468	2.3192e-168
DWCC	0.3102	1.2055e-265
CC	0.2037	7.8364e-115
KC	0.3481	0.0000e-00

Table 7: Kolmogorov-Smirnov statistic and p-value for all features in D_{all}.

3.2 Box-plots for the distribution of features

In this section we provide box-plots in all subsets for the empirical distributions of all features: **SCC** = Number of Strongly Connected Components; **LSCC** = Size of the Largest Strongly Connected Component; **WCC** = Number of Weakly Connected Components; **LWCC** = Size of the Largest Weakly Connected Component; **DWCC** = Diameter of the Largest Weakly Connected Components; **CC** = Average Clustering Coefficient; **KC** = Main K-Core Number.

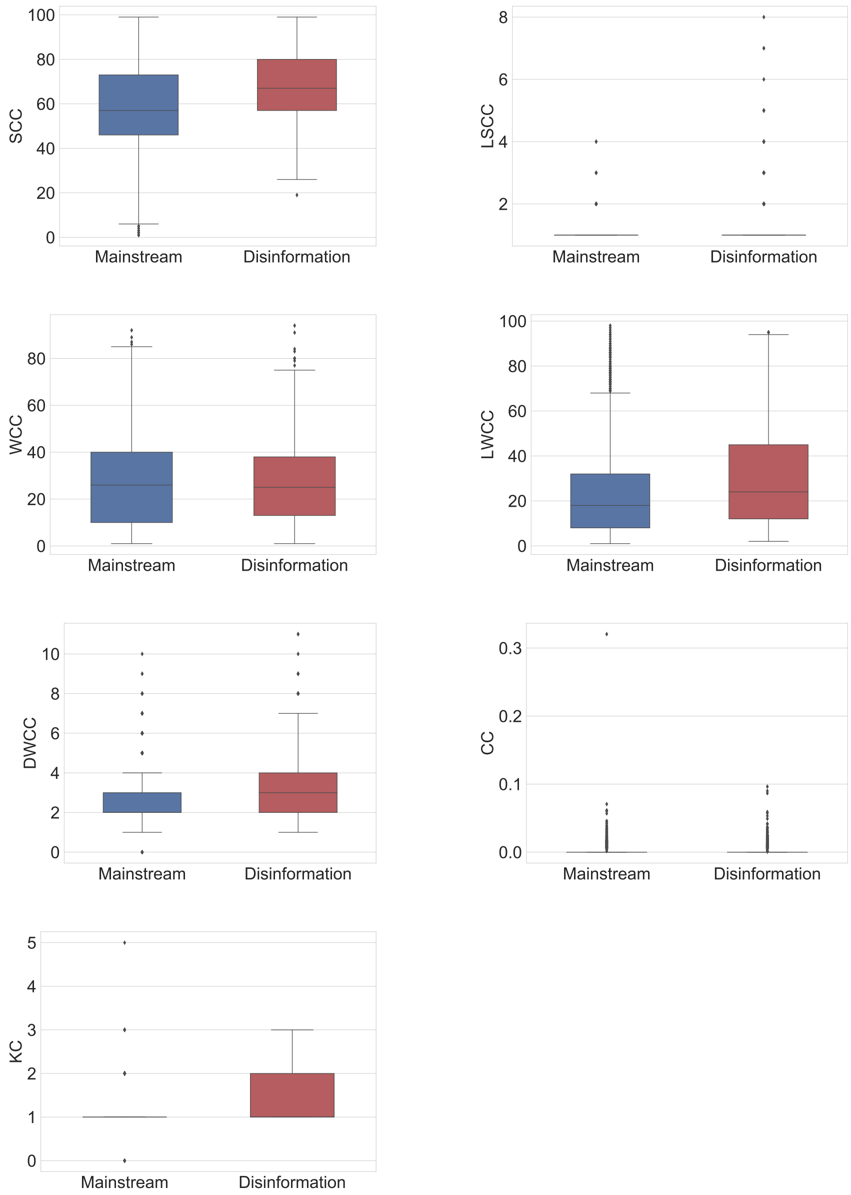

Figure 3: Box plots for all global network properties in $D_{[0,100)}$

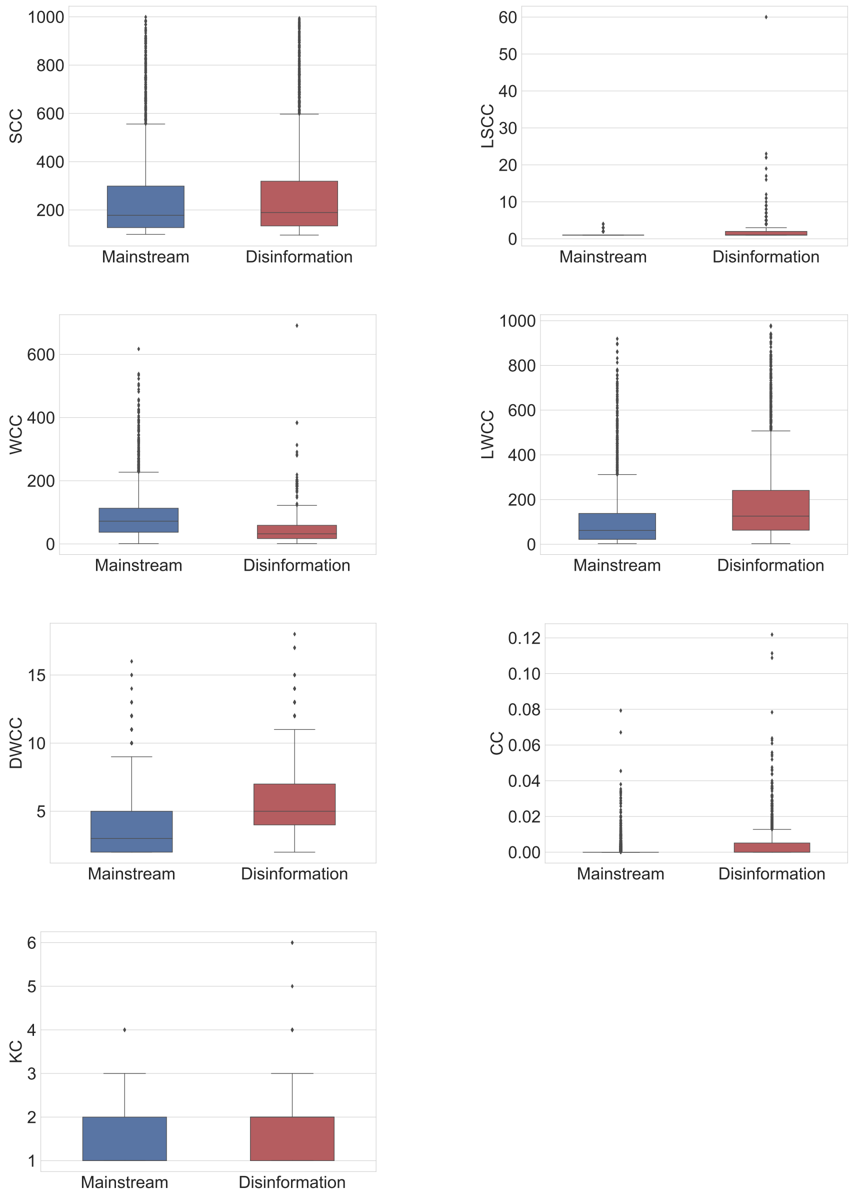

Figure 4: Box plots for all global network properties in $D_{[100,1000)}$

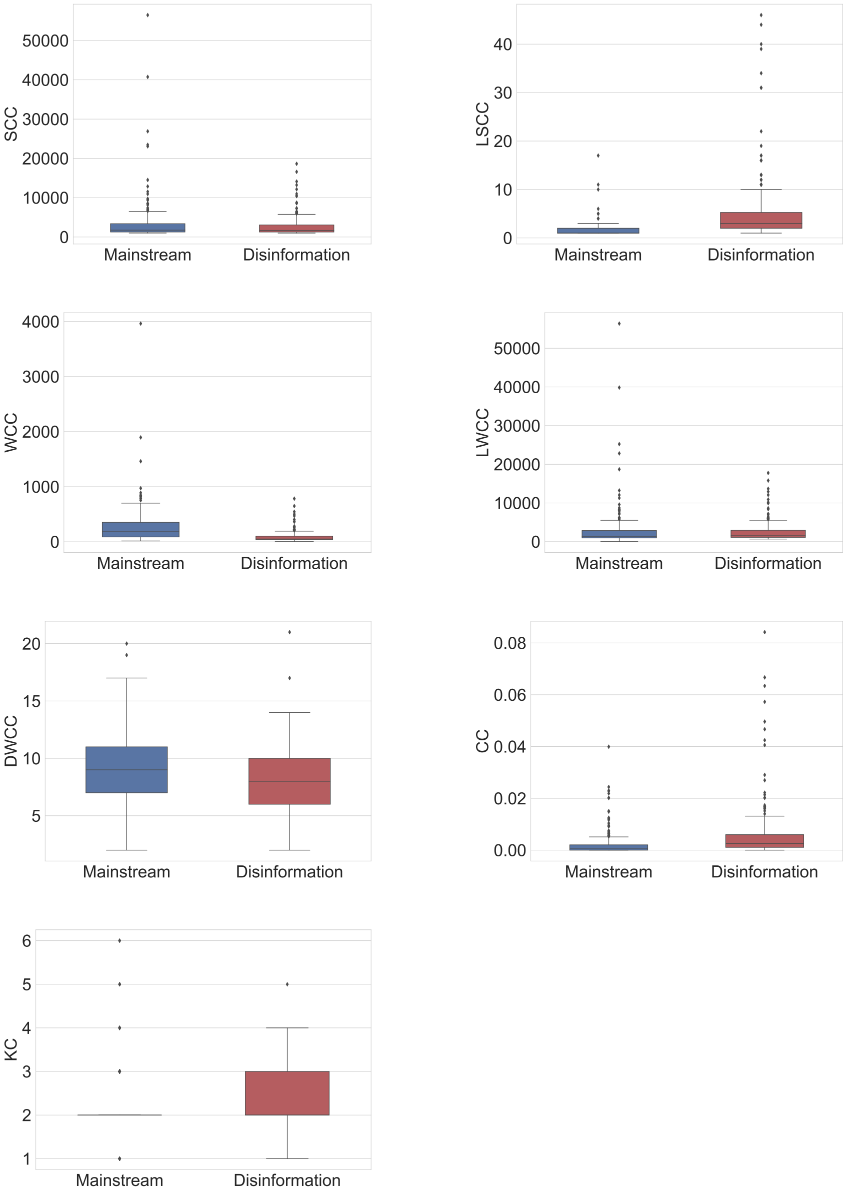

Figure 5: Box plots for all global network properties in $D_{[1000,+\infty)}$

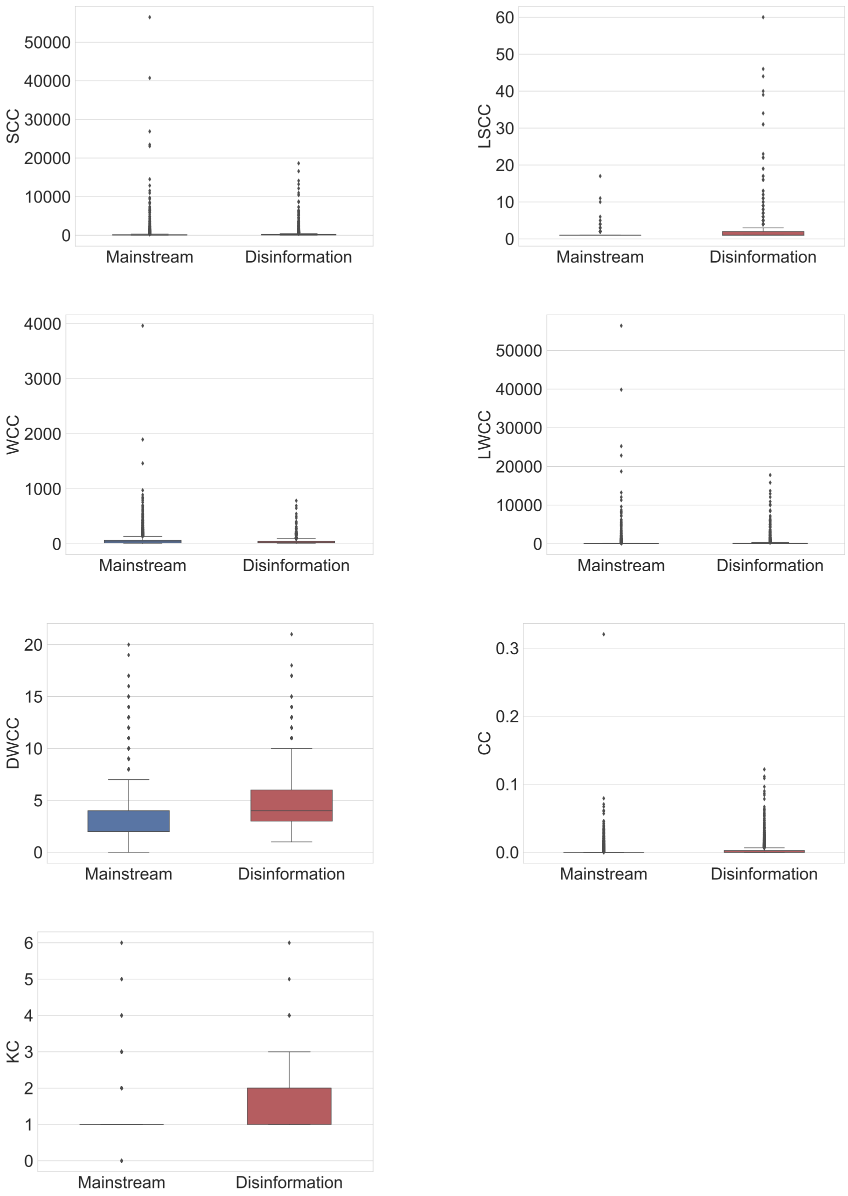

Figure 6: Box plots for all global network properties in D_{all}

3.3 Correlation Analysis

In this section we provide the Pearson pairwise correlation of all features in all subsets (including the size of networks) computed according to `pandas` Python package [11].

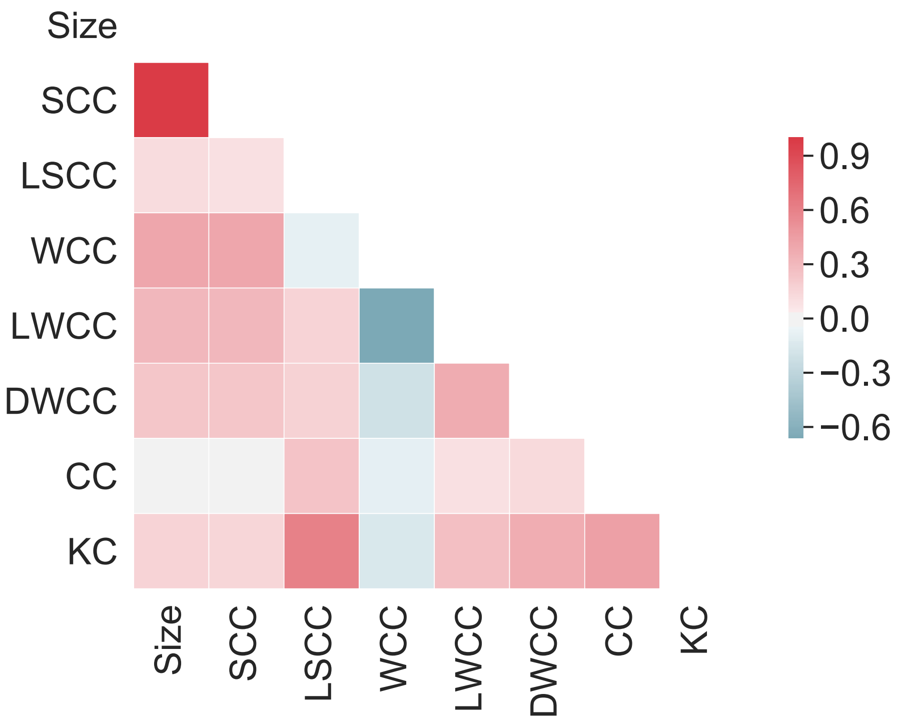

Figure 7: Correlation matrix for $D_{[0,100)}$.

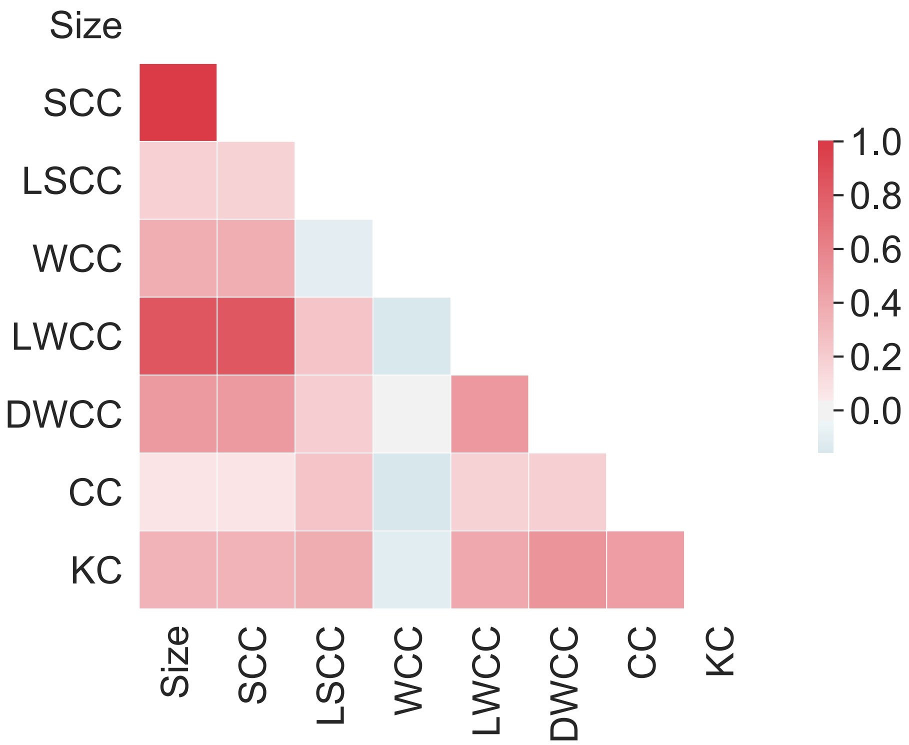

Figure 8: Correlation matrix for $D_{[100,1000)}$.

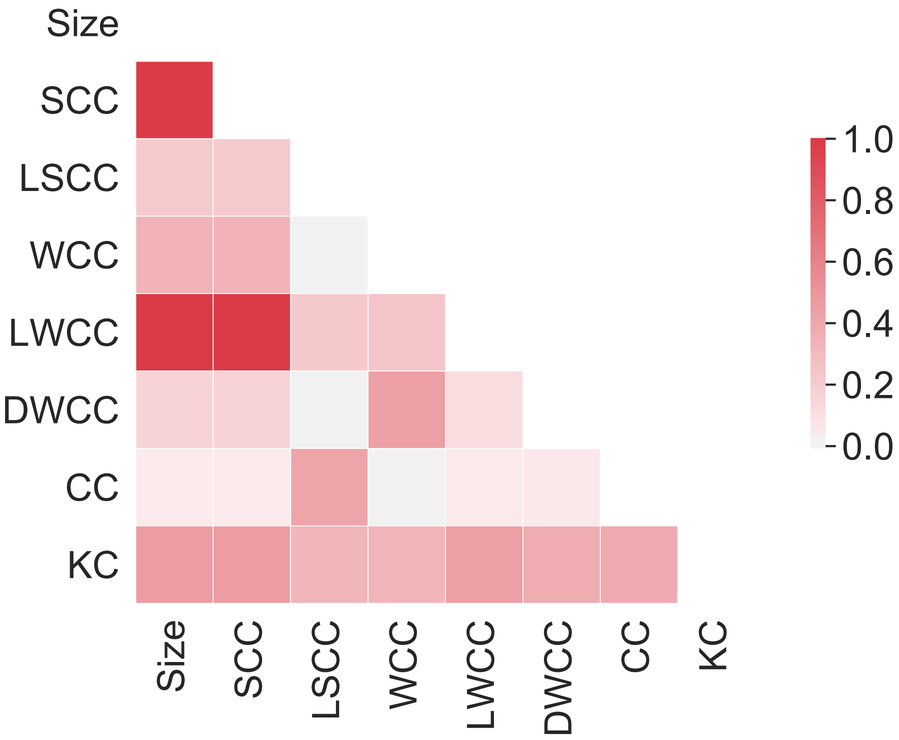

Figure 9: Correlation matrix for $D_{[1000,+\infty)}$.

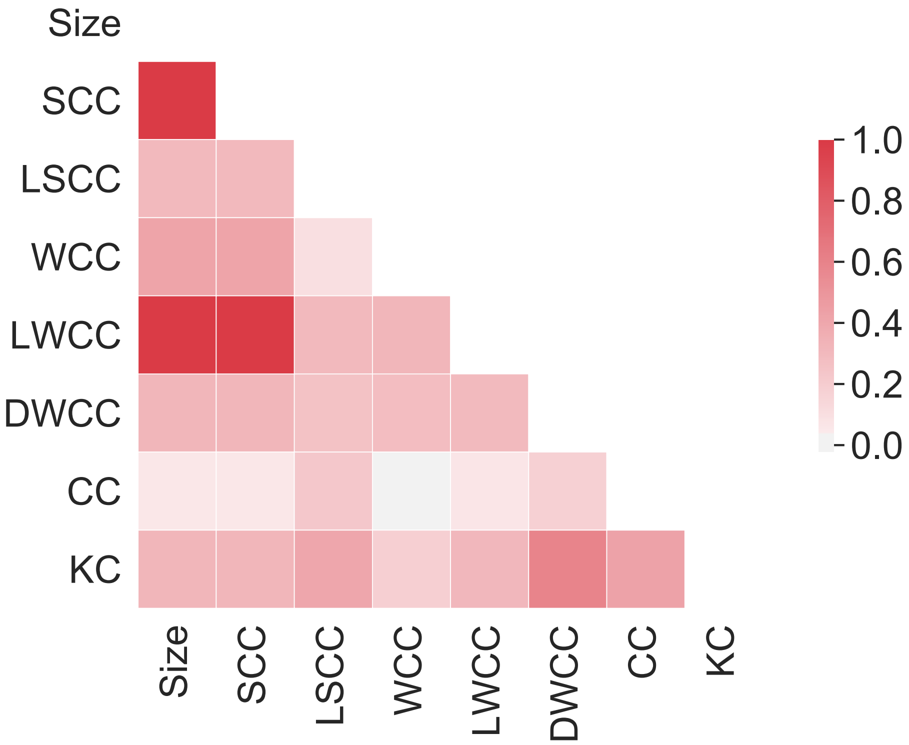

Figure 10: Correlation matrix for D_{all}.

4 Classification

4.1 Evaluation Metrics

Given two classes denoted as *positive* and *negative* we define:

- True Positives (TP) = number of actual positives which are recognized as positives.
- False Positives (FP) = number of actual negatives which are recognized as positives.
- True Negatives (TN) = number of actual negatives which are recognized as negatives.
- False Negatives (FN) = number of actual positives which are recognized as negatives.

We used the following evaluation metrics to assess the performances of different classifiers:

1. Precision $= \frac{TP}{(TP+FP)}$, the ability of a classifier not to label as positive a negative sample.
2. Recall $= \frac{TP}{(TP+FN)}$, the ability of a classifier to retrieve all positive samples.
3. F1-score $= 2\frac{precision \cdot recall}{precision+recall}$, the harmonic average of precision and recall.
4. Area Under the Receiver Operating Characteristic curve (AUROC); the Receiver Operating Characteristic (ROC) curve [6], which plots the TP rate versus the FP rate, shows the ability of a classifier to discriminate positive samples from negative ones as its threshold is varied; the AUROC value is in the range $[0, 1]$, with the random baseline classifier holding AUROC$= 0.5$ and the ideal perfect classifier AUROC$= 1$; thus larger AUROC values (and steeper ROCs) corresponds to better classifiers.

In all cases we computed so-called *macro* average–simple unweighted mean–of these metrics evaluated considering in turn both labels (misleading and mainstream) as positive class. We employed the `sklearn` Python package [14] for calculations.

To evaluate classifier performances we used cross validation, which is a statistical technique usually employed to estimate the performances of a predictive model on unseen data [5]. In our experiments we used the Stratified-Shuffle-Split approach, which involves generating a certain number of independent (random) train/test dataset splits where the same percentage for each class is preserved.

4.2 Classification results for Global Network Properties

In addition to classifiers specified in the main text, we also evaluated the following state-of-the-art classifiers: Support Vector Machine (SVM) with linear and RBF kernels, Gradient Boosting with exponential and deviance loss, Random Forest. We used the implementations available in the `sklearn` Python package [14] with default parameters (and no hyperparameter tuning). We provide evaluation results in tables S6 to S9.

	**Evaluation Metrics for $D_{[0,100)}$**			
**Classifier**	Recall	Precision	F1-Score	AUROC
SVC Linear	0.63 (sd 0.01)	0.73 (sd 0.03)	0.63 (sd 0.01)	0.72 (sd 0.03)
SVC RBF	0.68 (sd 0.02)	0.71 (sd 0.02)	0.69 (sd 0.02)	0.74 (sd 0.02)
Logistic Regression	0.65 (sd 0.01)	0.70 (sd 0.01)	0.65 (sd 0.01)	0.74 (sd 0.02)
Random Forest	0.68 (sd 0.02)	0.68 (sd 0.02)	0.68 (sd 0.02)	0.75 (sd 0.02)
K-NN (k=5)	0.66 (sd 0.02)	0.67 (sd 0.01)	0.67 (sd 0.02)	0.74 (sd 0.01)
K-NN (k=10)	0.67 (sd 0.02)	0.70 (sd 0.01)	0.67 (sd 0.02)	0.76 (sd 0.01)
K-NN (k=20)	0.68 (sd 0.02)	0.71 (sd 0.02)	0.69 (sd 0.02)	0.77 (sd 0.01)
K-NN (k=50)	0.68 (sd 0.01)	0.72 (sd 0.02)	0.69 (sd 0.01)	0.78 (sd 0.01)
Gradient Boosting (exponential)	0.69 (sd 0.01)	0.72 (sd 0.01)	0.70 (sd 0.01)	0.78 (sd 0.01)
Gradient Boosting (deviance)	0.69 (sd 0.01)	0.72 (sd 0.01)	0.69 (sd 0.01)	0.78 (sd 0.01)

Table 8: Classification metrics for all classifiers evaluated using global network properties in $D_{[0,100)}$.

	**Evaluation Metrics for $D_{[100,1000)}$**			
**Classifier**	Recall	Precision	F1-Score	AUROC
SVC Linear	0.75 (sd 0.01)	0.75 (sd 0.01)	0.75 (sd 0.01)	0.84 (sd 0.01)
SVC RBF	0.76 (sd 0.02)	0.76 (sd 0.02)	0.76 (sd 0.02)	0.82 (sd 0.02)
Logistic Regression	0.75 (sd 0.02)	0.76 (sd 0.01)	0.74 (sd 0.02)	0.85 (sd 0.02)
Random Forest	0.76 (sd 0.02)	0.76 (sd 0.02)	0.76 (sd 0.02)	0.85 (sd 0.02)
K-NN (k=5)	0.75 (sd 0.02)	0.75 (sd 0.02)	0.75 (sd 0.02)	0.83 (sd 0.02)
K-NN (k=10)	0.76 (sd 0.02)	0.76 (sd 0.02)	0.76 (sd 0.02)	0.85 (sd 0.02)
K-NN (k=20)	0.76 (sd 0.02)	0.76 (sd 0.02)	0.76 (sd 0.02)	0.85 (sd 0.01)
K-NN (k=50)	0.76 (sd 0.01)	0.76 (sd 0.01)	0.76 (sd 0.01)	0.86 (sd 0.01)
Gradient Boosting (exponential)	0.76 (sd 0.02)	0.76 (sd 0.02)	0.76 (sd 0.02)	0.86 (sd 0.02)
Gradient Boosting (deviance)	0.76 (sd 0.02)	0.76 (sd 0.02)	0.76 (sd 0.02)	0.86 (sd 0.02)

Table 9: Classification metrics for all classifiers evaluated using global network properties in $D_{[100,1000)}$.

	Evaluation Metrics for $D_{[1000,+\infty)}$			
**Classifier**	Recall	Precision	F1-Score	AUROC
SVC Linear	0.78 (sd 0.04)	0.82 (sd 0.05)	0.78 (sd 0.05)	0.89 (sd 0.04)
SVC RBF	0.73 (sd 0.06)	0.79 (sd 0.06)	0.73 (sd 0.07)	0.86 (sd 0.06)
Logistic Regression	0.85 (sd 0.06)	0.86 (sd 0.06)	0.85 (sd 0.06)	0.93 (sd 0.03)
Random Forest	0.84 (sd 0.07)	0.85 (sd 0.07)	0.84 (sd 0.07)	0.91 (sd 0.04)
K-NN (k=5)	0.80 (sd 0.05)	0.81 (sd 0.04)	0.81 (sd 0.05)	0.87 (sd 0.04)
K-NN (k=10)	0.84 (sd 0.04)	0.84 (sd 0.04)	0.84 (sd 0.04)	0.89 (sd 0.04)
K-NN (k=20)	0.82 (sd 0.05)	0.83 (sd 0.05)	0.82 (sd 0.05)	0.89 (sd 0.03)
K-NN (k=50)	0.79 (sd 0.06)	0.81 (sd 0.06)	0.79 (sd 0.06)	0.87 (sd 0.04)
Gradient Boosting (exponential)	0.81 (sd 0.05)	0.84 (sd 0.06)	0.82 (sd 0.05)	0.90 (sd 0.04)
Gradient Boosting (deviance)	0.82 (sd 0.06)	0.83 (sd 0.07)	0.82 (sd 0.06)	0.89 (sd 0.04)

Table 10: Classification metrics for all classifiers evaluated using global network properties in $D_{[1000,+\infty)}$.

	Evaluation Metrics for D_{all}			
**Classifier**	Recall	Precision	F1-Score	AUROC
SVC Linear	0.69 (sd 0.01)	0.72 (sd 0.02)	0.69 (sd 0.02)	0.77 (sd 0.01)
SVC RBF	0.72 (sd 0.01)	0.74 (sd 0.01)	0.72 (sd 0.01)	0.75 (sd 0.03)
Logistic Regression	0.71 (sd 0.02)	0.74 (sd 0.02)	0.71 (sd 0.02)	0.78 (sd 0.02)
Random Forest	0.65 (sd 0.03)	0.65 (sd 0.03)	0.65 (sd 0.03)	0.73 (sd 0.04)
K-NN (k=5)	0.69 (sd 0.02)	0.70 (sd 0.02)	0.69 (sd 0.02)	0.75 (sd 0.02)
K-NN (k=10)	0.70 (sd 0.01)	0.72 (sd 0.02)	0.70 (sd 0.01)	0.77 (sd 0.02)
K-NN (k=20)	0.71 (sd 0.01)	0.73 (sd 0.02)	0.71 (sd 0.01)	0.78 (sd 0.01)
K-NN (k=50)	0.72 (sd 0.01)	0.74 (sd 0.02)	0.72 (sd 0.01)	0.79 (sd 0.01)
Gradient Boosting (exponential)	0.69 (sd 0.04)	0.70 (sd 0.04)	0.69 (sd 0.04)	0.76 (sd 0.03)
Gradient Boosting (deviance)	0.67 (sd 0.05)	0.67 (sd 0.05)	0.67 (sd 0.05)	0.73 (sd 0.06)

Table 11: Classification metrics for all classifiers evaluated using global network properties in D_{all}.

4.3 Classification results for Centrality Measures and Network distances

Evaluation of all aforementioned centrality measures and distance networks are available in the attached files (respectively "centrality_results.csv" and "distances_results.csv").

4.4 Classification results for Global Network Properties with Sampling

We collected misleading stories in the period from 25th February 2019 to 18th March 2019, which was used for mainstream news. As resulting misleading networks were strongly imbalanced (1157 misleading networks vs 6878 mainstream networks), we used imblearn Python package [10] to apply three different sampling approaches:

1. Random Under Sampling: we uniformly randomly sampled individuals from the majority class (mainstream).

2. Random Over Sampling: we uniformly randomly sampled individuals from the minority class (misleading).

3. Balanced Classifiers: the `imblearn` package also provides a way to train boosting classifiers, in particular we chose Random Forest and AdaBoost, trained on samples which are balanced in the two classes.

We evaluated Precision, Recall, F1-Score and AUROC for all before mentioned classifiers on all subsets as it follows: for 1) and 2) we first split networks according to the number of nodes ($[0, 100)$, $[100, 1000)$, $[1000, +\infty)$, all) and then evaluated metrics with 10-fold stratified shuffle split on 100 different samples; we report accordingly the average value of metrics over all samples (tables S10 to S20). For what concerns 3) we simply evaluated both classifiers with 10-fold stratified shuffle split on the original collection.

	**Evaluation Metrics with Random Under Sampling for $D_{[0,100)}$**			
**Classifier**	Recall	Precision	F1-Score	AUROC
SVC Linear	0.68 (sd 0.00)	0.69 (sd 0.00)	0.68 (sd 0.00)	0.75 (sd 0.00)
SVC RBF	0.69 (sd 0.00)	0.69 (sd 0.00)	0.69 (sd 0.00)	0.75 (sd 0.00)
Logistic Regression	0.68 (sd 0.00)	0.68 (sd 0.00)	0.68 (sd 0.00)	0.75 (sd 0.00)
Random Forest	0.66 (sd 0.01)	0.66 (sd 0.01)	0.66 (sd 0.01)	0.74 (sd 0.00)
K-NN (N=5)	0.68 (sd 0.00)	0.68 (sd 0.00)	0.68 (sd 0.00)	0.74 (sd 0.00)
K-NN (N=10)	0.67 (sd 0.00)	0.67 (sd 0.00)	0.67 (sd 0.00)	0.75 (sd 0.00)
K-NN (N=20)	0.67 (sd 0.00)	0.67 (sd 0.00)	0.67 (sd 0.00)	0.76 (sd 0.00)
K-NN (N=50)	0.69 (sd 0.00)	0.69 (sd 0.00)	0.68 (sd 0.00)	0.78 (sd 0.00)
Gradient Boosting (exponential)	0.69 (sd 0.00)	0.69 (sd 0.00)	0.69 (sd 0.00)	0.77 (sd 0.00)
Gradient Boosting (deviance)	0.69 (sd 0.00)	0.69 (sd 0.00)	0.69 (sd 0.00)	0.76 (sd 0.00)

Table 12: Classification metrics for all classifiers evaluated using global network properties and random under sampling in $D_{[0,100)}$.

Classifier	Evaluation Metrics with Random Under Sampling for $D_{[100,1000)}$			
	Recall	Precision	F1-Score	AUROC
SVC Linear	0.75 (sd 0.00)	0.76 (sd 0.00)	0.75 (sd 0.00)	0.83 (sd 0.00)
SVC RBF	0.74 (sd 0.00)	0.74 (sd 0.00)	0.74 (sd 0.00)	0.78 (sd 0.00)
Logistic Regression	0.76 (sd 0.00)	0.76 (sd 0.00)	0.76 (sd 0.00)	0.85 (sd 0.00)
Random Forest	0.75 (sd 0.01)	0.75 (sd 0.01)	0.74 (sd 0.01)	0.83 (sd 0.00)
K-NN (N=5)	0.75 (sd 0.00)	0.75 (sd 0.00)	0.75 (sd 0.00)	0.81 (sd 0.00)
K-NN (N=10)	0.76 (sd 0.00)	0.76 (sd 0.00)	0.76 (sd 0.00)	0.84 (sd 0.00)
K-NN (N=20)	0.76 (sd 0.00)	0.76 (sd 0.00)	0.76 (sd 0.00)	0.85 (sd 0.00)
K-NN (N=50)	0.75 (sd 0.00)	0.75 (sd 0.00)	0.75 (sd 0.00)	0.84 (sd 0.00)
Gradient Boosting (exponential)	0.74 (sd 0.00)	0.74 (sd 0.00)	0.73 (sd 0.00)	0.84 (sd 0.00)
Gradient Boosting (deviance)	0.74 (sd 0.00)	0.74 (sd 0.00)	0.74 (sd 0.00)	0.83 (sd 0.00)

Table 13: Classification metrics for all classifiers evaluated using global network properties and random under sampling in $D_{[100,1000)}$.

Classifier	Evaluation Metrics with Random Under Sampling for $D_{[1000,+\infty)}$			
	Recall	Precision	F1-Score	AUROC
SVC Linear	0.64 (sd 0.00)	0.73 (sd 0.00)	0.59 (sd 0.00)	0.66 (sd 0.08)
SVC RBF	0.70 (sd 0.00)	0.72 (sd 0.00)	0.68 (sd 0.00)	0.79 (sd 0.00)
Logistic Regression	0.76 (sd 0.00)	0.77 (sd 0.00)	0.75 (sd 0.00)	0.82 (sd 0.00)
Random Forest	0.73 (sd 0.02)	0.74 (sd 0.02)	0.71 (sd 0.02)	0.79 (sd 0.01)
K-NN (N=5)	0.77 (sd 0.00)	0.80 (sd 0.00)	0.75 (sd 0.00)	0.84 (sd 0.00)
K-NN (N=10)	0.71 (sd 0.00)	0.77 (sd 0.00)	0.67 (sd 0.00)	0.83 (sd 0.00)
K-NN (N=20)	0.75 (sd 0.00)	0.80 (sd 0.00)	0.72 (sd 0.00)	0.85 (sd 0.00)
K-NN (N=50)	0.62 (sd 0.00)	0.66 (sd 0.00)	0.59 (sd 0.00)	0.62 (sd 0.00)
Gradient Boosting (exponential)	0.71 (sd 0.01)	0.72 (sd 0.01)	0.70 (sd 0.01)	0.75 (sd 0.01)
Gradient Boosting (deviance)	0.72 (sd 0.01)	0.74 (sd 0.01)	0.71 (sd 0.01)	0.76 (sd 0.01)

Table 14: Classification metrics for all classifiers evaluated using global network properties and random under sampling in $D_{[1000,+\infty)}$.

Classifier	Evaluation Metrics with Random Under Sampling for D_{all}			
	Recall	Precision	F1-Score	AUROC
SVC Linear	0.68 (sd 0.00)	0.70 (sd 0.00)	0.67 (sd 0.00)	0.75 (sd 0.00)
SVC RBF	0.70 (sd 0.00)	0.70 (sd 0.00)	0.70 (sd 0.00)	0.73 (sd 0.00)
Logistic Regression	0.69 (sd 0.00)	0.71 (sd 0.00)	0.68 (sd 0.00)	0.76 (sd 0.00)
Random Forest	0.59 (sd 0.00)	0.60 (sd 0.01)	0.57 (sd 0.00)	0.63 (sd 0.01)
K-NN (N=5)	0.67 (sd 0.00)	0.68 (sd 0.00)	0.67 (sd 0.00)	0.73 (sd 0.00)
K-NN (N=10)	0.70 (sd 0.00)	0.71 (sd 0.00)	0.69 (sd 0.00)	0.76 (sd 0.00)
K-NN (N=20)	0.69 (sd 0.00)	0.70 (sd 0.00)	0.69 (sd 0.00)	0.77 (sd 0.00)
K-NN (N=50)	0.71 (sd 0.00)	0.72 (sd 0.00)	0.71 (sd 0.00)	0.78 (sd 0.00)
Gradient Boosting (exponential)	0.62 (sd 0.00)	0.63 (sd 0.00)	0.61 (sd 0.01)	0.68 (sd 0.00)
Gradient Boosting (deviance)	0.61 (sd 0.00)	0.62 (sd 0.00)	0.59 (sd 0.01)	0.66 (sd 0.00)

Table 15: Classification metrics for all classifiers evaluated using global network properties and random under sampling in D_{all}.

Classifier	Evaluation Metrics with Random Over Sampling for $D_{[0,100)}$ Recall	Precision	F1-Score	AUROC
SVC Linear	0.67 (sd 0.00)	0.68 (sd 0.00)	0.67 (sd 0.00)	0.74 (sd 0.00)
SVC RBF	0.79 (sd 0.00)	0.79 (sd 0.00)	0.79 (sd 0.00)	0.86 (sd 0.00)
Logistic Regression	0.68 (sd 0.00)	0.68 (sd 0.00)	0.68 (sd 0.00)	0.74 (sd 0.00)
Random Forest	0.87 (sd 0.00)	0.87 (sd 0.00)	0.86 (sd 0.00)	0.94 (sd 0.00)
K-NN (N=5)	0.86 (sd 0.00)	0.88 (sd 0.00)	0.86 (sd 0.00)	0.92 (sd 0.00)
K-NN (N=10)	0.80 (sd 0.00)	0.81 (sd 0.00)	0.79 (sd 0.00)	0.88 (sd 0.00)
K-NN (N=20)	0.77 (sd 0.00)	0.77 (sd 0.00)	0.76 (sd 0.00)	0.85 (sd 0.00)
K-NN (N=50)	0.73 (sd 0.00)	0.73 (sd 0.00)	0.73 (sd 0.00)	0.82 (sd 0.00)
Gradient Boosting (exponential)	0.72 (sd 0.00)	0.72 (sd 0.00)	0.72 (sd 0.00)	0.82 (sd 0.00)
Gradient Boosting (deviance)	0.73 (sd 0.00)	0.73 (sd 0.00)	0.73 (sd 0.00)	0.82 (sd 0.00)

Table 16: Classification metrics for all classifiers evaluated using global network properties and random over sampling in $D_{[0,100)}$.

Classifier	Evaluation Metrics with Random Over Sampling for $D_{[100,1000)}$ Recall	Precision	F1-Score	AUROC
SVC Linear	0.76 (sd 0.00)	0.77 (sd 0.00)	0.76 (sd 0.00)	0.85 (sd 0.00)
SVC RBF	0.83 (sd 0.00)	0.84 (sd 0.00)	0.83 (sd 0.00)	0.89 (sd 0.00)
Logistic Regression	0.76 (sd 0.00)	0.76 (sd 0.00)	0.76 (sd 0.00)	0.86 (sd 0.00)
Random Forest	0.86 (sd 0.00)	0.87 (sd 0.00)	0.86 (sd 0.00)	0.93 (sd 0.00)
K-NN (N=5)	0.84 (sd 0.00)	0.84 (sd 0.00)	0.83 (sd 0.00)	0.90 (sd 0.00)
K-NN (N=10)	0.80 (sd 0.00)	0.80 (sd 0.00)	0.80 (sd 0.00)	0.89 (sd 0.00)
K-NN (N=20)	0.79 (sd 0.00)	0.79 (sd 0.00)	0.79 (sd 0.00)	0.88 (sd 0.00)
K-NN (N=50)	0.79 (sd 0.00)	0.79 (sd 0.00)	0.79 (sd 0.00)	0.87 (sd 0.00)
Gradient Boosting (exponential)	0.78 (sd 0.00)	0.79 (sd 0.00)	0.78 (sd 0.00)	0.88 (sd 0.00)
Gradient Boosting (deviance)	0.79 (sd 0.00)	0.79 (sd 0.00)	0.79 (sd 0.00)	0.88 (sd 0.00)

Table 17: Classification metrics for all classifiers evaluated using global network properties and random over sampling in $D_{[100,1000)}$.

Classifier	Evaluation Metrics with Random Over Sampling for $D_{[1000,+\infty)}$ Recall	Precision	F1-Score	AUROC
SVC Linear	0.74 (sd 0.00)	0.74 (sd 0.00)	0.74 (sd 0.00)	0.82 (sd 0.00)
SVC RBF	0.72 (sd 0.00)	0.76 (sd 0.00)	0.70 (sd 0.00)	0.86 (sd 0.00)
Logistic Regression	0.76 (sd 0.00)	0.77 (sd 0.00)	0.76 (sd 0.00)	0.82 (sd 0.00)
Random Forest	0.73 (sd 0.01)	0.80 (sd 0.01)	0.71 (sd 0.02)	0.87 (sd 0.01)
K-NN (N=5)	0.80 (sd 0.00)	0.80 (sd 0.00)	0.79 (sd 0.00)	0.87 (sd 0.00)
K-NN (N=10)	0.74 (sd 0.00)	0.74 (sd 0.00)	0.74 (sd 0.00)	0.84 (sd 0.00)
K-NN (N=20)	0.72 (sd 0.00)	0.73 (sd 0.00)	0.72 (sd 0.00)	0.84 (sd 0.00)
K-NN (N=50)	0.72 (sd 0.00)	0.73 (sd 0.00)	0.72 (sd 0.00)	0.83 (sd 0.00)
Gradient Boosting (exponential)	0.73 (sd 0.00)	0.76 (sd 0.00)	0.72 (sd 0.00)	0.85 (sd 0.00)
Gradient Boosting (deviance)	0.72 (sd 0.00)	0.75 (sd 0.01)	0.71 (sd 0.00)	0.84 (sd 0.00)

Table 18: Classification metrics for all classifiers evaluated using global network properties and random over sampling in $D_{[1000,+\infty)}$.

Classifier	Evaluation Metrics with Random Over Sampling for D_{all} Recall	Precision	F1-Score	AUROC
SVC Linear	0.70 (sd 0.00)	0.72 (sd 0.00)	0.69 (sd 0.00)	0.76 (sd 0.00)
SVC RBF	0.73 (sd 0.00)	0.75 (sd 0.00)	0.73 (sd 0.00)	0.78 (sd 0.00)
Logistic Regression	0.71 (sd 0.00)	0.72 (sd 0.00)	0.70 (sd 0.00)	0.77 (sd 0.00)
Random Forest	0.65 (sd 0.00)	0.70 (sd 0.01)	0.62 (sd 0.00)	0.71 (sd 0.00)
K-NN (N=5)	0.71 (sd 0.00)	0.72 (sd 0.00)	0.71 (sd 0.00)	0.75 (sd 0.00)
K-NN (N=10)	0.71 (sd 0.00)	0.71 (sd 0.00)	0.71 (sd 0.00)	0.77 (sd 0.00)
K-NN (N=20)	0.71 (sd 0.00)	0.72 (sd 0.00)	0.71 (sd 0.00)	0.78 (sd 0.00)
K-NN (N=50)	0.72 (sd 0.00)	0.72 (sd 0.00)	0.71 (sd 0.00)	0.79 (sd 0.00)
Gradient Boosting (exponential)	0.67 (sd 0.00)	0.68 (sd 0.00)	0.67 (sd 0.00)	0.74 (sd 0.00)
Gradient Boosting (deviance)	0.67 (sd 0.00)	0.67 (sd 0.00)	0.67 (sd 0.00)	0.72 (sd 0.00)

Table 19: Classification metrics for all classifiers evaluated using global network properties and random over sampling in D_{all}.

Classifier	Evaluation Metrics with Balanced Classifiers for $D_{[0,100)}$ Recall	Precision	F1-Score	AUROC
Balanced Random Forest	0.69 (sd 0.03)	0.59 (sd 0.01)	0.56 (sd 0.02)	0.78 (sd 0.03)
Balanced AdaBoost	0.69 (sd 0.05)	0.58 (sd 0.02)	0.57 (sd 0.03)	0.77 (sd 0.04)

Table 20: Classification metrics for all classifiers evaluated using global network properties and balanced classifiers in $D_{[0,100)}$.

Classifier	Evaluation Metrics with Balanced Classifiers for $D_{[100,1000)}$ Recall	Precision	F1-Score	AUROC
Balanced Random Forest	0.77 (sd 0.02)	0.69 (sd 0.02)	0.71 (sd 0.02)	0.86 (sd 0.02)
Balanced AdaBoost	0.75 (sd 0.03)	0.69 (sd 0.02)	0.70 (sd 0.03)	0.84 (sd 0.02)

Table 21: Classification metrics for all classifiers evaluated using global network properties and balanced classifiers in $D_{[100,1000)}$.

Classifier	Evaluation Metrics with Balanced Classifiers for $D_{[1000,+\infty)}$ Recall	Precision	F1-Score	AUROC
Balanced Random Forest	0.74 (sd 0.09)	0.69 (sd 0.12)	0.68 (sd 0.10)	0.82 (sd 0.11)
Balanced AdaBoost	0.73 (sd 0.11)	0.67 (sd 0.09)	0.68 (sd 0.10)	0.81 (sd 0.11)

Table 22: Classification metrics for all classifiers evaluated using global network properties and balanced classifiers in $D_{[1000,+\infty)}$.

Classifier	Evaluation Metrics with Balanced Classifiers for D_{all} Recall	Precision	F1-Score	AUROC
Balanced Random Forest	0.68 (sd 0.03)	0.61 (sd 0.01)	0.61 (sd 0.03)	0.75 (sd 0.05)
Balanced AdaBoost	0.69 (sd 0.04)	0.63 (sd 0.02)	0.63 (sd 0.02)	0.76 (sd 0.05)

Table 23: Classification metrics for all classifiers evaluated using global network properties and balanced classifiers in D_{all}.

5 Classification performances taking into account bias labels on sources

In this section we provide classification performance results when taking into account also the bias of news domains. Labels are obtained as in [4]. We show results, in terms of Precision, Recall, F1-Score and AUROC, concerning several combinations of training and test sets, as specified in the caption of tables 24 to 55.

They overall show similar classification performances compared to the general case discussed in the main text.

	**Evaluation Metrics for Classifiers in D_{all}**			
**Classifier**	Recall	Precision	F1-Score	AUROC
SVC Linear	0.66 (sd $1.73E-02$)	0.79 (sd $1.26E-02$)	0.68 (sd $1.97E-02$)	0.73 (sd $2.45E-02$)
SVC RBF	0.66 (sd $1.28E-02$)	0.79 (sd $1.48E-02$)	0.68 (sd $1.48E-02$)	0.67 (sd $3.28E-02$)
Logistic Regression	0.66 (sd $2.12E-02$)	0.78 (sd $2.45E-02$)	0.68 (sd $2.48E-02$)	0.77 (sd $1.62E-02$)
Random Forest	0.66 (sd $1.92E-02$)	0.74 (sd $2.64E-02$)	0.68 (sd $2.12E-02$)	0.74 (sd $1.84E-02$)
K-NN (N=5)	0.67 (sd $1.37E-02$)	0.74 (sd $1.67E-02$)	0.69 (sd $1.37E-02$)	0.72 (sd $1.53E-02$)
K-NN (N=10)	0.67 (sd $1.44E-02$)	0.78 (sd $1.06E-02$)	0.69 (sd $1.56E-02$)	0.74 (sd $1.21E-02$)
K-NN (N=20)	0.68 (sd $1.36E-02$)	0.79 (sd $1.58E-02$)	0.70 (sd $1.49E-02$)	0.76 (sd $1.09E-02$)
K-NN (N=50)	0.67 (sd $1.34E-02$)	0.79 (sd $1.44E-02$)	0.70 (sd $1.44E-02$)	0.78 (sd $1.14E-02$)
Gradient Boosting (exponential)	0.66 (sd $2.35E-02$)	0.80 (sd $1.89E-02$)	0.68 (sd $2.72E-02$)	0.75 (sd $2.39E-02$)
Gradient Boosting (deviance)	0.66 (sd $2.68E-02$)	0.80 (sd $1.67E-02$)	0.68 (sd $3.07E-02$)	0.76 (sd $2.57E-02$)
Balanced RF	0.68 (sd $2.04E-02$)	0.69 (sd $3.02E-02$)	0.68 (sd $2.46E-02$)	0.75 (sd $1.72E-02$)
Balanced ADABOOST	0.69 (sd $3.89E-02$)	0.69 (sd $2.13E-02$)	0.68 (sd $3.25E-02$)	0.77 (sd $2.70E-02$)

Table 24: Classification metrics for balanced classifiers evaluated using global network properties in D_{all}. Training and test data include all sources except Breitbart.com and Politicususa.com.

	**Evaluation Metrics for Classifiers in $D_{[0,100]}$**			
**Classifier**	Recall	Precision	F1-Score	AUROC
SVC Linear	0.59 (sd $1.84E-02$)	0.80 (sd $3.67E-02$)	0.60 (sd $2.70E-02$)	0.65 (sd $6.58E-02$)
SVC RBF	0.57 (sd $2.36E-02$)	0.70 (sd $4.02E-02$)	0.57 (sd $3.51E-02$)	0.69 (sd $3.31E-02$)
Logistic Regression	0.60 (sd $2.22E-02$)	0.76 (sd $5.53E-02$)	0.61 (sd $3.06E-02$)	0.75 (sd $2.71E-02$)
Random Forest	0.62 (sd $1.82E-02$)	0.69 (sd $1.89E-02$)	0.64 (sd $2.01E-02$)	0.75 (sd $2.18E-02$)
K-NN (N=5)	0.63 (sd $2.46E-02$)	0.70 (sd $2.90E-02$)	0.65 (sd $2.75E-02$)	0.71 (sd $2.63E-02$)
K-NN (N=10)	0.62 (sd $2.05E-02$)	0.75 (sd $3.78E-02$)	0.64 (sd $2.59E-02$)	0.74 (sd $2.13E-02$)
K-NN (N=20)	0.61 (sd $1.85E-02$)	0.75 (sd $3.53E-02$)	0.64 (sd $2.42E-02$)	0.76 (sd $2.42E-02$)
K-NN (N=50)	0.60 (sd $2.53E-02$)	0.76 (sd $4.04E-02$)	0.62 (sd $3.40E-02$)	0.77 (sd $2.43E-02$)
Gradient Boosting (exponential)	0.60 (sd $2.07E-02$)	0.75 (sd $3.01E-02$)	0.62 (sd $2.82E-02$)	0.77 (sd $1.78E-02$)
Gradient Boosting (deviance)	0.60 (sd $2.41E-02$)	0.74 (sd $4.27E-02$)	0.62 (sd $3.16E-02$)	0.77 (sd $1.90E-02$)
Balanced RF	0.69 (sd $2.23E-02$)	0.63 (sd $1.48E-02$)	0.63 (sd $1.61E-02$)	0.77 (sd $2.28E-02$)
Balanced ADABOOST	0.69 (sd $2.02E-02$)	0.64 (sd $1.36E-02$)	0.64 (sd $1.62E-02$)	0.77 (sd $1.59E-02$)

Table 25: Classification metrics for balanced classifiers evaluated using global network properties in $D_{[0,100]}$. Training and test data include all sources except Breitbart.com and Politicususa.com.

Evaluation Metrics for Classifiers in $D_{[100,1000]}$				
**Classifier**	Recall	Precision	F1-Score	AUROC
SVC Linear	0.70 (sd $1.50E-02$)	0.78 (sd $1.54E-02$)	0.72 (sd $1.60E-02$)	0.83 (sd $1.50E-02$)
SVC RBF	0.72 (sd $1.79E-02$)	0.78 (sd $2.39E-02$)	0.74 (sd $1.93E-02$)	0.79 (sd $1.55E-02$)
Logistic Regression	0.72 (sd $9.61E-03$)	0.79 (sd $1.92E-02$)	0.73 (sd $1.08E-02$)	0.85 (sd $2.02E-02$)
Random Forest	0.75 (sd $1.29E-02$)	0.78 (sd $1.81E-02$)	0.76 (sd $1.40E-02$)	0.85 (sd $1.24E-02$)
K-NN (N=5)	0.74 (sd $1.93E-02$)	0.76 (sd $2.15E-02$)	0.75 (sd $1.93E-02$)	0.81 (sd $1.68E-02$)
K-NN (N=10)	0.74 (sd $1.49E-02$)	0.78 (sd $2.24E-02$)	0.75 (sd $1.60E-02$)	0.84 (sd $1.67E-02$)
K-NN (N=20)	0.75 (sd $1.26E-02$)	0.80 (sd $1.98E-02$)	0.77 (sd $1.42E-02$)	0.84 (sd $1.63E-02$)
K-NN (N=50)	0.75 (sd $1.74E-02$)	0.79 (sd $2.45E-02$)	0.76 (sd $1.90E-02$)	0.85 (sd $2.10E-02$)
Gradient Boosting (exponential)	0.75 (sd $1.80E-02$)	0.81 (sd $2.84E-02$)	0.77 (sd $2.02E-02$)	0.86 (sd $1.59E-02$)
Gradient Boosting (deviance)	0.75 (sd $1.81E-02$)	0.81 (sd $2.65E-02$)	0.77 (sd $1.99E-02$)	0.86 (sd $1.37E-02$)
Balanced RF	0.77 (sd $1.41E-02$)	0.75 (sd $1.41E-02$)	0.76 (sd $1.41E-02$)	0.85 (sd $1.23E-02$)
Balanced ADABOOST	0.75 (sd $2.14E-02$)	0.73 (sd $2.84E-02$)	0.74 (sd $2.65E-02$)	0.84 (sd $1.29E-02$)

Table 26: Classification metrics for all classifiers evaluated using global network properties in $D_{[100,1000]}$. Training and test data include all sources except Breitbart.com and Politicususa.com.

Evaluation Metrics for Classifiers in $D_{[1000,+\infty]}$				
**Classifier**	Recall	Precision	F1-Score	AUROC
SVC Linear	0.63 (sd $7.04E-02$)	0.78 (sd $1.30E-01$)	0.63 (sd $9.24E-02$)	0.91 (sd $3.85E-02$)
SVC RBF	0.69 (sd $1.09E-01$)	0.78 (sd $1.10E-01$)	0.69 (sd $1.29E-01$)	0.88 (sd $5.82E-02$)
Logistic Regression	0.82 (sd $9.51E-02$)	0.85 (sd $7.86E-02$)	0.82 (sd $9.48E-02$)	0.92 (sd $4.72E-02$)
Random Forest	0.77 (sd $9.65E-02$)	0.81 (sd $9.39E-02$)	0.78 (sd $9.48E-02$)	0.90 (sd $6.17E-02$)
K-NN (N=5)	0.77 (sd $4.86E-02$)	0.79 (sd $5.68E-02$)	0.77 (sd $5.06E-02$)	0.86 (sd $5.65E-02$)
K-NN (N=10)	0.73 (sd $9.11E-02$)	0.78 (sd $7.48E-02$)	0.73 (sd $9.23E-02$)	0.87 (sd $4.83E-02$)
K-NN (N=20)	0.72 (sd $8.42E-02$)	0.77 (sd $6.48E-02$)	0.73 (sd $8.65E-02$)	0.86 (sd $4.45E-02$)
K-NN (N=50)	0.68 (sd $4.86E-02$)	0.80 (sd $7.21E-02$)	0.69 (sd $5.51E-02$)	0.83 (sd $4.50E-02$)
Gradient Boosting (exponential)	0.77 (sd $7.78E-02$)	0.77 (sd $7.86E-02$)	0.75 (sd $7.35E-02$)	0.88 (sd $7.61E-02$)
Gradient Boosting (deviance)	0.75 (sd $1.07E-01$)	0.75 (sd $9.24E-02$)	0.73 (sd $9.48E-02$)	0.87 (sd $8.85E-02$)
Balanced RF	0.81 (sd $7.91E-02$)	0.82 (sd $7.75E-02$)	0.81 (sd $7.37E-02$)	0.91 (sd $5.24E-02$)
Balanced ADABOOST	0.78 (sd $8.95E-02$)	0.80 (sd $1.05E-01$)	0.77 (sd $9.70E-02$)	0.86 (sd $7.23E-02$)

Table 27: Classification metrics for all classifiers evaluated using global network properties in $D_{[1000,+\infty]}$. Training and test data include all sources except Breitbart.com and Politicususa.com.

Evaluation Metrics for Classifiers in D_{all}				
**Classifier**	Recall	Precision	F1-Score	AUROC
SVC Linear	0.67 (sd $4.97E-03$)	0.78 (sd $1.04E-02$)	0.66 (sd $6.13E-03$)	0.76 (sd $8.18E-03$)
SVC RBF	0.72 (sd $1.05E-02$)	0.75 (sd $1.43E-02$)	0.72 (sd $1.12E-02$)	0.74 (sd $2.86E-02$)
Logistic Regression	0.70 (sd $1.07E-02$)	0.75 (sd $1.25E-02$)	0.70 (sd $1.20E-02$)	0.77 (sd $7.45E-03$)
Random Forest	0.67 (sd $4.40E-02$)	0.68 (sd $4.87E-02$)	0.67 (sd $4.54E-02$)	0.74 (sd $3.93E-02$)
K-NN (N=5)	0.69 (sd $9.10E-03$)	0.71 (sd $1.33E-02$)	0.70 (sd $8.93E-03$)	0.75 (sd $1.51E-02$)
K-NN (N=10)	0.70 (sd $1.33E-02$)	0.74 (sd $1.50E-02$)	0.70 (sd $1.41E-02$)	0.77 (sd $1.64E-02$)
K-NN (N=20)	0.71 (sd $8.43E-03$)	0.75 (sd $1.33E-02$)	0.72 (sd $8.93E-03$)	0.79 (sd $1.30E-02$)
K-NN (N=50)	0.72 (sd $9.63E-03$)	0.75 (sd $1.43E-02$)	0.73 (sd $1.02E-02$)	0.80 (sd $1.34E-02$)
Gradient Boosting (exponential)	0.69 (sd $3.64E-02$)	0.72 (sd $4.46E-02$)	0.69 (sd $3.81E-02$)	0.77 (sd $3.60E-02$)
Gradient Boosting (deviance)	0.68 (sd $4.48E-02$)	0.69 (sd $5.36E-02$)	0.68 (sd $4.75E-02$)	0.75 (sd $5.50E-02$)
Balanced RF	0.67 (sd $3.61E-02$)	0.68 (sd $2.95E-02$)	0.67 (sd $3.44E-02$)	0.75 (sd $3.64E-02$)
Balanced ADABOOST	0.69 (sd $4.36E-02$)	0.73 (sd $1.46E-02$)	0.69 (sd $4.76E-02$)	0.76 (sd $4.25E-02$)

Table 28: Classification metrics for all classifiers evaluated using global network properties in D_{all}. Training and test data include all sources except Politicususa.com.

Evaluation Metrics for Classifiers in $D_{[0,100]}$				
**Classifier**	Recall	Precision	F1-Score	AUROC
SVC Linear	0.59 (sd 1.81$E-02$)	0.77 (sd 3.07$E-02$)	0.58 (sd 2.67$E-02$)	0.70 (sd 4.67$E-02$)
SVC RBF	0.68 (sd 1.77$E-02$)	0.72 (sd 2.21$E-02$)	0.69 (sd 1.92$E-02$)	0.74 (sd 3.15$E-02$)
Logistic Regression	0.64 (sd 2.02$E-02$)	0.72 (sd 2.80$E-02$)	0.65 (sd 2.38$E-02$)	0.75 (sd 2.52$E-02$)
Random Forest	0.67 (sd 2.54$E-02$)	0.69 (sd 2.81$E-02$)	0.68 (sd 2.66$E-02$)	0.77 (sd 3.07$E-02$)
K-NN (N=5)	0.66 (sd 1.98$E-02$)	0.68 (sd 2.32$E-02$)	0.67 (sd 2.09$E-02$)	0.74 (sd 2.40$E-02$)
K-NN (N=10)	0.66 (sd 1.98$E-02$)	0.71 (sd 2.58$E-02$)	0.67 (sd 2.20$E-02$)	0.76 (sd 2.14$E-02$)
K-NN (N=20)	0.67 (sd 2.07$E-02$)	0.72 (sd 2.73$E-02$)	0.68 (sd 2.29$E-02$)	0.77 (sd 2.73$E-02$)
K-NN (N=50)	0.67 (sd 1.63$E-02$)	0.73 (sd 1.99$E-02$)	0.68 (sd 1.79$E-02$)	0.78 (sd 2.53$E-02$)
Gradient Boosting (exponential)	0.68 (sd 1.45$E-02$)	0.73 (sd 1.74$E-02$)	0.69 (sd 1.56$E-02$)	0.79 (sd 2.36$E-02$)
Gradient Boosting (deviance)	0.68 (sd 1.60$E-02$)	0.73 (sd 2.02$E-02$)	0.69 (sd 1.74$E-02$)	0.79 (sd 2.51$E-02$)
Balanced RF	0.70 (sd 2.36$E-02$)	0.68 (sd 2.19$E-02$)	0.68 (sd 2.31$E-02$)	0.78 (sd 2.82$E-02$)
Balanced ADABOOST	0.69 (sd 2.44$E-02$)	0.68 (sd 2.28$E-02$)	0.68 (sd 2.39$E-02$)	0.78 (sd 2.46$E-02$)

Table 29: Classification metrics for all classifiers evaluated using global network properties in $D_{[0,100]}$. Training and test data include all sources except Politicususa.com.

Evaluation Metrics for Classifiers in $D_{[100,1000]}$				
**Classifier**	Recall	Precision	F1-Score	AUROC
SVC Linear	0.76 (sd 2.07$E-02$)	0.76 (sd 2.16$E-02$)	0.76 (sd 2.08$E-02$)	0.85 (sd 1.56$E-02$)
SVC RBF	0.76 (sd 2.03$E-02$)	0.76 (sd 1.99$E-02$)	0.76 (sd 2.04$E-02$)	0.81 (sd 2.21$E-02$)
Logistic Regression	0.75 (sd 2.13$E-02$)	0.77 (sd 2.27$E-02$)	0.75 (sd 2.17$E-02$)	0.85 (sd 1.71$E-02$)
Random Forest	0.76 (sd 1.65$E-02$)	0.77 (sd 1.72$E-02$)	0.76 (sd 1.66$E-02$)	0.85 (sd 1.59$E-02$)
K-NN (N=5)	0.75 (sd 2.05$E-02$)	0.75 (sd 2.06$E-02$)	0.75 (sd 2.04$E-02$)	0.82 (sd 2.22$E-02$)
K-NN (N=10)	0.76 (sd 1.28$E-02$)	0.77 (sd 1.29$E-02$)	0.76 (sd 1.30$E-02$)	0.84 (sd 1.69$E-02$)
K-NN (N=20)	0.76 (sd 1.45$E-02$)	0.76 (sd 1.40$E-02$)	0.76 (sd 1.46$E-02$)	0.85 (sd 1.89$E-02$)
K-NN (N=50)	0.77 (sd 1.36$E-02$)	0.77 (sd 1.36$E-02$)	0.77 (sd 1.36$E-02$)	0.85 (sd 1.67$E-02$)
Gradient Boosting (exponential)	0.76 (sd 1.42$E-02$)	0.77 (sd 1.60$E-02$)	0.76 (sd 1.43$E-02$)	0.86 (sd 1.70$E-02$)
Gradient Boosting (deviance)	0.77 (sd 1.75$E-02$)	0.77 (sd 1.92$E-02$)	0.77 (sd 1.76$E-02$)	0.86 (sd 1.86$E-02$)
Balanced RF	0.77 (sd 1.32$E-02$)	0.77 (sd 1.39$E-02$)	0.77 (sd 1.33$E-02$)	0.85 (sd 1.62$E-02$)
Balanced ADABOOST	0.76 (sd 1.91$E-02$)	0.76 (sd 1.91$E-02$)	0.76 (sd 1.92$E-02$)	0.85 (sd 1.94$E-02$)

Table 30: Classification metrics for all classifiers evaluated using global network properties in $D_{[100,1000]}$. Training and test data include all sources except Politicususa.com.

Evaluation Metrics for Classifiers in $D_{[1000,+\infty]}$				
**Classifier**	Recall	Precision	F1-Score	AUROC
SVC Linear	0.81 (sd 3.88$E-02$)	0.82 (sd 3.80$E-02$)	0.81 (sd 3.93$E-02$)	0.90 (sd 3.13$E-02$)
SVC RBF	0.78 (sd 5.02$E-02$)	0.79 (sd 5.05$E-02$)	0.78 (sd 5.10$E-02$)	0.88 (sd 5.17$E-02$)
Logistic Regression	0.84 (sd 4.48$E-02$)	0.85 (sd 4.61$E-02$)	0.84 (sd 4.49$E-02$)	0.93 (sd 2.75$E-02$)
Random Forest	0.83 (sd 5.68$E-02$)	0.84 (sd 5.27$E-02$)	0.83 (sd 5.83$E-02$)	0.92 (sd 3.31$E-02$)
K-NN (N=5)	0.83 (sd 6.50$E-02$)	0.83 (sd 6.53$E-02$)	0.83 (sd 6.51$E-02$)	0.89 (sd 6.36$E-02$)
K-NN (N=10)	0.81 (sd 6.52$E-02$)	0.81 (sd 6.61$E-02$)	0.81 (sd 6.53$E-02$)	0.89 (sd 5.04$E-02$)
K-NN (N=20)	0.81 (sd 4.77$E-02$)	0.81 (sd 4.69$E-02$)	0.81 (sd 4.80$E-02$)	0.87 (sd 4.27$E-02$)
K-NN (N=50)	0.77 (sd 7.91$E-02$)	0.77 (sd 7.96$E-02$)	0.77 (sd 7.97$E-02$)	0.86 (sd 4.53$E-02$)
Gradient Boosting (exponential)	0.82 (sd 4.72$E-02$)	0.83 (sd 4.63$E-02$)	0.82 (sd 4.76$E-02$)	0.91 (sd 3.21$E-02$)
Gradient Boosting (deviance)	0.81 (sd 5.15$E-02$)	0.82 (sd 5.26$E-02$)	0.81 (sd 5.16$E-02$)	0.89 (sd 4.01$E-02$)
Balanced RF	0.84 (sd 5.61$E-02$)	0.85 (sd 5.70$E-02$)	0.84 (sd 5.64$E-02$)	0.93 (sd 3.62$E-02$)
Balanced ADABOOST	0.79 (sd 6.24$E-02$)	0.80 (sd 5.85$E-02$)	0.79 (sd 6.57$E-02$)	0.88 (sd 4.92$E-02$)

Table 31: Classification metrics for all classifiers evaluated using global network properties in $D_{[1000,+\infty]}$. Training and test data include all sources except Politicususa.com.

Classifier	Recall	Precision	F1-Score	AUROC
	**Evaluation Metrics for Classifiers in D_{all}**			
SVC Linear	0.66 (sd $1.10E-02$)	0.79 (sd $1.68E-02$)	0.67 (sd $1.35E-02$)	0.75 (sd $1.84E-02$)
SVC RBF	0.69 (sd $1.18E-02$)	0.78 (sd $1.56E-02$)	0.71 (sd $1.33E-02$)	0.72 (sd $2.91E-02$)
Logistic Regression	0.68 (sd $8.65E-03$)	0.77 (sd $2.08E-02$)	0.70 (sd $9.99E-03$)	0.78 (sd $1.59E-02$)
Random Forest	0.66 (sd $2.79E-02$)	0.75 (sd $1.73E-02$)	0.67 (sd $3.19E-02$)	0.74 (sd $3.82E-02$)
K-NN (N=5)	0.69 (sd $1.41E-02$)	0.73 (sd $1.05E-02$)	0.70 (sd $1.42E-02$)	0.74 (sd $1.66E-02$)
K-NN (N=10)	0.69 (sd $1.09E-02$)	0.78 (sd $7.69E-03$)	0.71 (sd $1.18E-02$)	0.77 (sd $1.74E-02$)
K-NN (N=20)	0.70 (sd $1.24E-02$)	0.78 (sd $1.46E-02$)	0.71 (sd $1.39E-02$)	0.78 (sd $1.94E-02$)
K-NN (N=50)	0.70 (sd $1.50E-02$)	0.78 (sd $1.35E-02$)	0.71 (sd $1.62E-02$)	0.79 (sd $1.54E-02$)
Gradient Boosting (exponential)	0.66 (sd $2.37E-02$)	0.76 (sd $3.15E-02$)	0.67 (sd $2.82E-02$)	0.75 (sd $4.30E-02$)
Gradient Boosting (deviance)	0.64 (sd $3.63E-02$)	0.68 (sd $7.26E-02$)	0.65 (sd $4.17E-02$)	0.73 (sd $5.15E-02$)
Balanced RF	0.68 (sd $2.95E-02$)	0.72 (sd $2.48E-02$)	0.69 (sd $2.52E-02$)	0.75 (sd $3.95E-02$)
Balanced ADABOOST	0.67 (sd $4.00E-02$)	0.72 (sd $1.59E-02$)	0.67 (sd $3.81E-02$)	0.74 (sd $4.37E-02$)

Table 32: Classification metrics for all classifiers evaluated using global network properties in D_{all}. Training and test data include all sources except Breitbart.com.

Classifier	Recall	Precision	F1-Score	AUROC
	**Evaluation Metrics for Classifiers in $D_{[0,100]}$**			
SVC Linear	0.59 (sd $1.54E-02$)	0.80 (sd $3.72E-02$)	0.59 (sd $2.26E-02$)	0.66 (sd $6.02E-02$)
SVC RBF	0.61 (sd $2.55E-02$)	0.71 (sd $2.98E-02$)	0.62 (sd $3.35E-02$)	0.72 (sd $2.35E-02$)
Logistic Regression	0.61 (sd $1.81E-02$)	0.74 (sd $3.39E-02$)	0.62 (sd $2.34E-02$)	0.75 (sd $1.72E-02$)
Random Forest	0.64 (sd $2.10E-02$)	0.69 (sd $2.89E-02$)	0.66 (sd $2.38E-02$)	0.75 (sd $2.12E-02$)
K-NN (N=5)	0.64 (sd $2.43E-02$)	0.68 (sd $2.67E-02$)	0.65 (sd $2.66E-02$)	0.72 (sd $2.24E-02$)
K-NN (N=10)	0.63 (sd $1.49E-02$)	0.73 (sd $2.33E-02$)	0.65 (sd $1.82E-02$)	0.75 (sd $2.28E-02$)
K-NN (N=20)	0.64 (sd $1.47E-02$)	0.74 (sd $2.50E-02$)	0.66 (sd $1.80E-02$)	0.77 (sd $2.06E-02$)
K-NN (N=50)	0.63 (sd $1.31E-02$)	0.74 (sd $2.96E-02$)	0.64 (sd $1.66E-02$)	0.77 (sd $1.63E-02$)
Gradient Boosting (exponential)	0.63 (sd $2.38E-02$)	0.75 (sd $3.59E-02$)	0.64 (sd $2.95E-02$)	0.78 (sd $1.24E-02$)
Gradient Boosting (deviance)	0.63 (sd $2.62E-02$)	0.74 (sd $3.55E-02$)	0.65 (sd $3.21E-02$)	0.79 (sd $1.43E-02$)
Balanced RF	0.69 (sd $2.21E-02$)	0.65 (sd $1.72E-02$)	0.65 (sd $1.76E-02$)	0.77 (sd $1.96E-02$)
Balanced ADABOOST	0.68 (sd $1.36E-02$)	0.65 (sd $9.66E-03$)	0.66 (sd $9.60E-03$)	0.77 (sd $1.31E-02$)

Table 33: Classification metrics for all classifiers evaluated using global network properties in $D_{[0,100]}$. Training and test data include all sources except Breitbart.com.

Classifier	Recall	Precision	F1-Score	AUROC
	**Evaluation Metrics for Classifiers in $D_{[100,1000]}$**			
SVC Linear	0.71 (sd $1.87E-02$)	0.80 (sd $2.12E-02$)	0.72 (sd $2.13E-02$)	0.84 (sd $1.65E-02$)
SVC RBF	0.75 (sd $1.48E-02$)	0.78 (sd $1.24E-02$)	0.76 (sd $1.43E-02$)	0.80 (sd $2.05E-02$)
Logistic Regression	0.75 (sd $1.49E-02$)	0.78 (sd $1.97E-02$)	0.75 (sd $1.59E-02$)	0.85 (sd $1.60E-02$)
Random Forest	0.76 (sd $1.81E-02$)	0.77 (sd $1.98E-02$)	0.76 (sd $1.84E-02$)	0.85 (sd $1.30E-02$)
K-NN (N=5)	0.75 (sd $2.15E-02$)	0.76 (sd $2.18E-02$)	0.75 (sd $2.15E-02$)	0.82 (sd $1.60E-02$)
K-NN (N=10)	0.75 (sd $1.93E-02$)	0.78 (sd $1.78E-02$)	0.76 (sd $1.95E-02$)	0.84 (sd $1.47E-02$)
K-NN (N=20)	0.75 (sd $1.30E-02$)	0.78 (sd $1.08E-02$)	0.76 (sd $1.29E-02$)	0.85 (sd $1.59E-02$)
K-NN (N=50)	0.76 (sd $1.29E-02$)	0.79 (sd $1.38E-02$)	0.77 (sd $1.31E-02$)	0.85 (sd $1.42E-02$)
Gradient Boosting (exponential)	0.76 (sd $1.90E-02$)	0.80 (sd $1.19E-02$)	0.76 (sd $1.90E-02$)	0.86 (sd $1.26E-02$)
Gradient Boosting (deviance)	0.76 (sd $1.69E-02$)	0.80 (sd $1.36E-02$)	0.77 (sd $1.71E-02$)	0.86 (sd $1.21E-02$)
Balanced RF	0.76 (sd $1.74E-02$)	0.76 (sd $1.65E-02$)	0.76 (sd $1.69E-02$)	0.85 (sd $1.38E-02$)
Balanced ADABOOST	0.76 (sd $1.71E-02$)	0.75 (sd $1.88E-02$)	0.76 (sd $1.80E-02$)	0.85 (sd $1.23E-02$)

Table 34: Classification metrics for all classifiers evaluated using global network properties in $D_{[100,1000]}$. Training and test data include all sources except Breitbart.com.

Classifier	Recall	Precision	F1-Score	AUROC
SVC Linear	0.75 (sd $5.88E-02$)	0.82 (sd $4.93E-02$)	0.76 (sd $6.45E-02$)	0.92 (sd $4.12E-02$)
SVC RBF	0.78 (sd $3.95E-02$)	0.83 (sd $5.38E-02$)	0.79 (sd $3.99E-02$)	0.89 (sd $5.37E-02$)
Logistic Regression	0.86 (sd $5.21E-02$)	0.85 (sd $5.70E-02$)	0.85 (sd $5.50E-02$)	0.93 (sd $3.47E-02$)
Random Forest	0.82 (sd $4.27E-02$)	0.83 (sd $3.78E-02$)	0.82 (sd $4.11E-02$)	0.90 (sd $3.15E-02$)
K-NN (N=5)	0.84 (sd $6.35E-02$)	0.84 (sd $5.90E-02$)	0.84 (sd $6.27E-02$)	0.91 (sd $4.30E-02$)
K-NN (N=10)	0.82 (sd $6.96E-02$)	0.83 (sd $6.69E-02$)	0.82 (sd $7.01E-02$)	0.90 (sd $4.34E-02$)
K-NN (N=20)	0.81 (sd $7.36E-02$)	0.82 (sd $7.33E-02$)	0.82 (sd $7.24E-02$)	0.89 (sd $4.66E-02$)
K-NN (N=50)	0.76 (sd $8.04E-02$)	0.79 (sd $7.58E-02$)	0.77 (sd $8.38E-02$)	0.88 (sd $5.60E-02$)
Gradient Boosting (exponential)	0.84 (sd $5.35E-02$)	0.84 (sd $5.29E-02$)	0.84 (sd $5.33E-02$)	0.89 (sd $4.31E-02$)
Gradient Boosting (deviance)	0.84 (sd $5.65E-02$)	0.83 (sd $5.63E-02$)	0.83 (sd $5.64E-02$)	0.89 (sd $4.59E-02$)
Balanced RF	0.83 (sd $5.38E-02$)	0.83 (sd $4.89E-02$)	0.83 (sd $5.03E-02$)	0.90 (sd $4.04E-02$)
Balanced ADABOOST	0.80 (sd $6.18E-02$)	0.80 (sd $6.23E-02$)	0.79 (sd $6.34E-02$)	0.90 (sd $3.41E-02$)

Evaluation Metrics for Classifiers in $D_{[1000,+\infty]}$

Table 35: Classification metrics for all classifiers evaluated using global network properties in $D_{[1000,+\infty]}$.Training and test data include all sources except Breitbart.com.

Evaluation Metrics for Classifiers in D_{all}

Classifier	Recall	Precision	F1-Score	AUROC
SVC Linear	0.71 (sd $1.23E-02$)	0.71 (sd $1.15E-02$)	0.69 (sd $1.41E-02$)	0.78 (sd $1.30E-02$)
SVC RBF	0.72 (sd $1.32E-02$)	0.72 (sd $1.37E-02$)	0.72 (sd $1.37E-02$)	0.77 (sd $1.45E-02$)
Logistic Regression	0.71 (sd $3.08E-02$)	0.71 (sd $3.06E-02$)	0.70 (sd $2.72E-02$)	0.78 (sd $1.54E-02$)
Random Forest	0.67 (sd $2.38E-02$)	0.68 (sd $2.48E-02$)	0.67 (sd $2.50E-02$)	0.77 (sd $2.63E-02$)
K-NN (N=5)	0.69 (sd $2.22E-02$)	0.69 (sd $1.82E-02$)	0.69 (sd $2.05E-02$)	0.76 (sd $1.85E-02$)
K-NN (N=10)	0.70 (sd $2.16E-02$)	0.70 (sd $2.04E-02$)	0.70 (sd $1.83E-02$)	0.78 (sd $1.49E-02$)
K-NN (N=20)	0.72 (sd $1.57E-02$)	0.72 (sd $1.39E-02$)	0.71 (sd $1.39E-02$)	0.80 (sd $1.14E-02$)
K-NN (N=50)	0.72 (sd $1.63E-02$)	0.72 (sd $1.29E-02$)	0.72 (sd $1.47E-02$)	0.81 (sd $1.12E-02$)
Gradient Boosting (exponential)	0.71 (sd $2.08E-02$)	0.71 (sd $2.10E-02$)	0.71 (sd $2.20E-02$)	0.79 (sd $2.45E-02$)
Gradient Boosting (deviance)	0.70 (sd $2.67E-02$)	0.70 (sd $2.74E-02$)	0.70 (sd $2.69E-02$)	0.78 (sd $3.27E-02$)
Balanced RF	0.69 (sd $1.73E-02$)	0.69 (sd $1.55E-02$)	0.68 (sd $2.17E-02$)	0.78 (sd $2.21E-02$)
Balanced ADABOOST	0.71 (sd $2.08E-02$)	0.72 (sd $1.34E-02$)	0.70 (sd $3.02E-02$)	0.78 (sd $3.44E-02$)

Table 36: Classification metrics for all classifiers evaluated using global network properties in D_{all}. Mainstream training and test data include only Left sources.

Evaluation Metrics for Classifiers in $D_{[0,100]}$

Classifier	Recall	Precision	F1-Score	AUROC
SVC Linear	0.68 (sd $1.55E-02$)	0.69 (sd $1.58E-02$)	0.67 (sd $1.64E-02$)	0.74 (sd $1.98E-02$)
SVC RBF	0.69 (sd $1.71E-02$)	0.69 (sd $1.66E-02$)	0.69 (sd $1.74E-02$)	0.75 (sd $2.01E-02$)
Logistic Regression	0.67 (sd $2.14E-02$)	0.68 (sd $2.27E-02$)	0.67 (sd $2.15E-02$)	0.75 (sd $1.86E-02$)
Random Forest	0.68 (sd $1.87E-02$)	0.68 (sd $1.87E-02$)	0.68 (sd $1.87E-02$)	0.77 (sd $2.14E-02$)
K-NN (N=5)	0.68 (sd $2.15E-02$)	0.68 (sd $2.13E-02$)	0.68 (sd $2.15E-02$)	0.75 (sd $2.13E-02$)
K-NN (N=10)	0.68 (sd $1.70E-02$)	0.69 (sd $1.66E-02$)	0.68 (sd $1.75E-02$)	0.77 (sd $1.91E-02$)
K-NN (N=20)	0.69 (sd $1.97E-02$)	0.69 (sd $1.99E-02$)	0.69 (sd $1.99E-02$)	0.78 (sd $1.90E-02$)
K-NN (N=50)	0.69 (sd $1.24E-02$)	0.69 (sd $1.24E-02$)	0.69 (sd $1.25E-02$)	0.78 (sd $1.44E-02$)
Gradient Boosting (exponential)	0.69 (sd $1.78E-02$)	0.70 (sd $1.73E-02$)	0.69 (sd $1.81E-02$)	0.79 (sd $1.66E-02$)
Gradient Boosting (deviance)	0.69 (sd $1.83E-02$)	0.70 (sd $1.79E-02$)	0.69 (sd $1.86E-02$)	0.79 (sd $1.71E-02$)
Balanced RF	0.68 (sd $1.83E-02$)	0.68 (sd $1.82E-02$)	0.68 (sd $1.83E-02$)	0.78 (sd $1.85E-02$)
Balanced ADABOOST	0.68 (sd $2.45E-02$)	0.69 (sd $2.42E-02$)	0.68 (sd $2.46E-02$)	0.77 (sd $1.90E-02$)

Table 37: Classification metrics for all classifiers evaluated using global network properties in $D_{[0,100]}$. Mainstream training and test data include only Left sources.

Classifier	Recall	Precision	F1-Score	AUROC
SVC Linear	0.76 (sd $1.99E-02$)	0.77 (sd $1.82E-02$)	0.76 (sd $1.88E-02$)	0.87 (sd $8.59E-03$)
SVC RBF	0.76 (sd $1.70E-02$)	0.78 (sd $1.70E-02$)	0.77 (sd $1.66E-02$)	0.82 (sd $1.04E-02$)
Logistic Regression	0.78 (sd $2.01E-02$)	0.77 (sd $1.92E-02$)	0.77 (sd $1.97E-02$)	0.87 (sd $1.14E-02$)
Random Forest	0.76 (sd $1.66E-02$)	0.77 (sd $1.68E-02$)	0.77 (sd $1.59E-02$)	0.87 (sd $9.46E-03$)
K-NN (N=5)	0.76 (sd $1.38E-02$)	0.76 (sd $1.58E-02$)	0.76 (sd $1.37E-02$)	0.84 (sd $1.29E-02$)
K-NN (N=10)	0.78 (sd $1.94E-02$)	0.78 (sd $1.84E-02$)	0.78 (sd $1.88E-02$)	0.86 (sd $1.29E-02$)
K-NN (N=20)	0.77 (sd $1.57E-02$)	0.78 (sd $1.67E-02$)	0.77 (sd $1.56E-02$)	0.87 (sd $8.59E-03$)
K-NN (N=50)	0.76 (sd $1.57E-02$)	0.78 (sd $1.54E-02$)	0.77 (sd $1.53E-02$)	0.87 (sd $1.06E-02$)
Gradient Boosting (exponential)	0.76 (sd $1.17E-02$)	0.78 (sd $1.11E-02$)	0.77 (sd $1.06E-02$)	0.88 (sd $9.11E-03$)
Gradient Boosting (deviance)	0.77 (sd $1.46E-02$)	0.78 (sd $1.43E-02$)	0.77 (sd $1.37E-02$)	0.88 (sd $9.62E-03$)
Balanced RF	0.79 (sd $1.63E-02$)	0.77 (sd $1.58E-02$)	0.77 (sd $1.68E-02$)	0.88 (sd $9.83E-03$)
Balanced ADABOOST	0.78 (sd $1.31E-02$)	0.77 (sd $1.26E-02$)	0.77 (sd $1.29E-02$)	0.87 (sd $7.09E-03$)

Evaluation Metrics for Classifiers in $D_{[100,1000]}$

Table 38: Classification metrics for all classifiers evaluated using global network properties in $D_{[100,1000]}$. Mainstream training and test data include only Left sources.

Evaluation Metrics for Classifiers in $D_{[1000,+\infty]}$

Classifier	Recall	Precision	F1-Score	AUROC
SVC Linear	0.66 (sd $3.75E-02$)	0.80 (sd $5.22E-02$)	0.67 (sd $4.56E-02$)	0.87 (sd $6.16E-02$)
SVC RBF	0.73 (sd $5.56E-02$)	0.80 (sd $4.68E-02$)	0.74 (sd $6.18E-02$)	0.83 (sd $7.35E-02$)
Logistic Regression	0.82 (sd $5.44E-02$)	0.84 (sd $4.29E-02$)	0.82 (sd $4.72E-02$)	0.90 (sd $4.17E-02$)
Random Forest	0.78 (sd $5.66E-02$)	0.79 (sd $5.76E-02$)	0.78 (sd $4.90E-02$)	0.88 (sd $4.88E-02$)
K-NN (N=5)	0.80 (sd $5.61E-02$)	0.80 (sd $5.72E-02$)	0.80 (sd $5.31E-02$)	0.86 (sd $4.04E-02$)
K-NN (N=10)	0.83 (sd $4.08E-02$)	0.82 (sd $4.66E-02$)	0.82 (sd $4.29E-02$)	0.89 (sd $3.85E-02$)
K-NN (N=20)	0.79 (sd $5.37E-02$)	0.82 (sd $5.19E-02$)	0.80 (sd $5.31E-02$)	0.87 (sd $4.07E-02$)
K-NN (N=50)	0.74 (sd $6.77E-02$)	0.81 (sd $4.64E-02$)	0.75 (sd $7.43E-02$)	0.86 (sd $5.24E-02$)
Gradient Boosting (exponential)	0.82 (sd $7.23E-02$)	0.83 (sd $6.45E-02$)	0.82 (sd $6.87E-02$)	0.88 (sd $5.75E-02$)
Gradient Boosting (deviance)	0.81 (sd $7.32E-02$)	0.82 (sd $7.01E-02$)	0.81 (sd $6.92E-02$)	0.88 (sd $5.38E-02$)
Balanced RF	0.79 (sd $4.60E-02$)	0.78 (sd $2.84E-02$)	0.77 (sd $3.57E-02$)	0.89 (sd $4.78E-02$)
Balanced ADABOOST	0.82 (sd $5.75E-02$)	0.81 (sd $4.58E-02$)	0.81 (sd $5.06E-02$)	0.88 (sd $4.34E-02$)

Table 39: Classification metrics for all classifiers evaluated using global network properties in $D_{[1000,+\infty]}$. Mainstream training and test data include only Left sources.

Evaluation Metrics for Classifiers in D_{all}

Classifier	Recall	Precision	F1-Score	AUROC
SVC Linear	0.50 (sd $0.00E+00$)	0.41 (sd $5.55E-17$)	0.45 (sd $0.00E+00$)	0.73 (sd $2.78E-02$)
SVC RBF	0.58 (sd $1.49E-02$)	0.75 (sd $6.44E-02$)	0.59 (sd $2.20E-02$)	0.67 (sd $3.00E-02$)
Logistic Regression	0.55 (sd $2.20E-02$)	0.78 (sd $7.45E-02$)	0.54 (sd $3.69E-02$)	0.78 (sd $1.67E-02$)
Random Forest	0.60 (sd $2.96E-02$)	0.64 (sd $3.78E-02$)	0.61 (sd $2.84E-02$)	0.75 (sd $2.61E-02$)
K-NN (N=5)	0.63 (sd $4.41E-02$)	0.65 (sd $3.29E-02$)	0.63 (sd $4.28E-02$)	0.73 (sd $2.98E-02$)
K-NN (N=10)	0.63 (sd $4.41E-02$)	0.65 (sd $2.71E-02$)	0.63 (sd $4.20E-02$)	0.76 (sd $2.07E-02$)
K-NN (N=20)	0.62 (sd $3.78E-02$)	0.69 (sd $4.63E-02$)	0.63 (sd $4.17E-02$)	0.77 (sd $1.80E-02$)
K-NN (N=50)	0.59 (sd $3.52E-02$)	0.72 (sd $4.89E-02$)	0.61 (sd $4.79E-02$)	0.79 (sd $1.56E-02$)
Gradient Boosting (exponential)	0.59 (sd $3.34E-02$)	0.69 (sd $5.25E-02$)	0.60 (sd $3.75E-02$)	0.78 (sd $2.94E-02$)
Gradient Boosting (deviance)	0.58 (sd $2.47E-02$)	0.69 (sd $5.39E-02$)	0.59 (sd $3.21E-02$)	0.77 (sd $2.94E-02$)
Balanced RF	0.71 (sd $1.72E-02$)	0.63 (sd $1.09E-02$)	0.59 (sd $4.05E-02$)	0.77 (sd $2.55E-02$)
Balanced ADABOOST	0.69 (sd $2.55E-02$)	0.62 (sd $1.60E-02$)	0.57 (sd $6.00E-02$)	0.74 (sd $4.06E-02$)

Table 40: Classification metrics for all classifiers evaluated using global network properties in D_{all}. Mainstream training and test data include only Right sources.

Evaluation Metrics for Classifiers in $D_{[0,100]}$

Classifier	Recall	Precision	F1-Score	AUROC
SVC Linear	0.50 (sd $0.00E+00$)	0.38 (sd $5.55E-17$)	0.43 (sd $0.00E+00$)	0.61 (sd $1.16E-01$)
SVC RBF	0.62 (sd $1.15E-02$)	0.82 (sd $3.82E-02$)	0.64 (sd $1.59E-02$)	0.61 (sd $3.27E-02$)
Logistic Regression	0.60 (sd $1.38E-02$)	0.82 (sd $2.23E-02$)	0.61 (sd $2.07E-02$)	0.74 (sd $2.04E-02$)
Random Forest	0.64 (sd $1.81E-02$)	0.71 (sd $1.59E-02$)	0.65 (sd $1.93E-02$)	0.75 (sd $1.61E-02$)
K-NN (N=5)	0.63 (sd $2.40E-02$)	0.69 (sd $2.99E-02$)	0.64 (sd $2.81E-02$)	0.71 (sd $1.72E-02$)
K-NN (N=10)	0.64 (sd $2.62E-02$)	0.72 (sd $3.40E-02$)	0.66 (sd $3.05E-02$)	0.74 (sd $2.45E-02$)
K-NN (N=20)	0.63 (sd $1.84E-02$)	0.76 (sd $2.96E-02$)	0.65 (sd $2.34E-02$)	0.75 (sd $2.30E-02$)
K-NN (N=50)	0.62 (sd $1.66E-02$)	0.80 (sd $4.21E-02$)	0.64 (sd $2.25E-02$)	0.76 (sd $1.63E-02$)
Gradient Boosting (exponential)	0.62 (sd $1.32E-02$)	0.80 (sd $2.89E-02$)	0.64 (sd $1.79E-02$)	0.77 (sd $2.40E-02$)
Gradient Boosting (deviance)	0.62 (sd $1.53E-02$)	0.78 (sd $4.21E-02$)	0.64 (sd $1.99E-02$)	0.77 (sd $2.38E-02$)
Balanced RF	0.68 (sd $1.73E-02$)	0.64 (sd $1.43E-02$)	0.64 (sd $1.87E-02$)	0.76 (sd $1.73E-02$)
Balanced ADABOOST	0.69 (sd $1.55E-02$)	0.64 (sd $1.19E-02$)	0.63 (sd $1.77E-02$)	0.76 (sd $1.78E-02$)

Table 41: Classification metrics for all classifiers evaluated using global network properties in $D_{[0,100]}$. Mainstream training and test data include only Right sources.

Evaluation Metrics for Classifiers in $D_{[100,1000]}$

Classifier	Recall	Precision	F1-Score	AUROC
SVC Linear	0.50 (sd $0.00E+00$)	0.43 (sd $5.55E-17$)	0.46 (sd $0.00E+00$)	0.84 (sd $2.69E-02$)
SVC RBF	0.58 (sd $2.10E-02$)	0.83 (sd $6.85E-02$)	0.60 (sd $3.29E-02$)	0.71 (sd $4.60E-02$)
Logistic Regression	0.62 (sd $4.71E-02$)	0.79 (sd $9.21E-02$)	0.64 (sd $4.76E-02$)	0.85 (sd $2.99E-02$)
Random Forest	0.64 (sd $3.01E-02$)	0.75 (sd $4.58E-02$)	0.67 (sd $3.62E-02$)	0.84 (sd $2.37E-02$)
K-NN (N=5)	0.62 (sd $3.66E-02$)	0.70 (sd $4.72E-02$)	0.64 (sd $4.15E-02$)	0.79 (sd $3.20E-02$)
K-NN (N=10)	0.63 (sd $3.16E-02$)	0.72 (sd $2.93E-02$)	0.66 (sd $3.41E-02$)	0.82 (sd $2.42E-02$)
K-NN (N=20)	0.61 (sd $3.03E-02$)	0.76 (sd $6.50E-02$)	0.64 (sd $3.85E-02$)	0.83 (sd $2.30E-02$)
K-NN (N=50)	0.61 (sd $3.06E-02$)	0.80 (sd $7.98E-02$)	0.64 (sd $4.25E-02$)	0.83 (sd $2.26E-02$)
Gradient Boosting (exponential)	0.61 (sd $2.42E-02$)	0.79 (sd $6.35E-02$)	0.64 (sd $3.34E-02$)	0.84 (sd $2.53E-02$)
Gradient Boosting (deviance)	0.61 (sd $2.72E-02$)	0.76 (sd $6.48E-02$)	0.64 (sd $3.69E-02$)	0.84 (sd $2.27E-02$)
Balanced RF	0.76 (sd $1.78E-02$)	0.64 (sd $8.26E-03$)	0.64 (sd $1.22E-02$)	0.85 (sd $2.22E-02$)
Balanced ADABOOST	0.75 (sd $2.83E-02$)	0.64 (sd $1.37E-02$)	0.65 (sd $1.92E-02$)	0.84 (sd $2.78E-02$)

Table 42: Classification metrics for all classifiers evaluated using global network properties in $D_{[100,1000]}$. Mainstream training and test data include only Right sources.

Evaluation Metrics for Classifiers in $D_{[1000,+\infty]}$

Classifier	Recall	Precision	F1-Score	AUROC
SVC Linear	0.50 (sd $0.00E+00$)	0.44 (sd $5.55E-17$)	0.47 (sd $0.00E+00$)	0.87 (sd $9.27E-02$)
SVC RBF	0.53 (sd $1.01E-01$)	0.50 (sd $1.61E-01$)	0.51 (sd $1.26E-01$)	0.70 (sd $2.03E-01$)
Logistic Regression	0.55 (sd $1.08E-01$)	0.58 (sd $2.11E-01$)	0.55 (sd $1.26E-01$)	0.86 (sd $1.03E-01$)
Random Forest	0.60 (sd $1.38E-01$)	0.66 (sd $2.32E-01$)	0.61 (sd $1.70E-01$)	0.81 (sd $7.79E-02$)
K-NN (N=5)	0.58 (sd $1.07E-01$)	0.59 (sd $1.77E-01$)	0.58 (sd $1.25E-01$)	0.68 (sd $1.07E-01$)
K-NN (N=10)	0.56 (sd $1.18E-01$)	0.60 (sd $2.41E-01$)	0.56 (sd $1.53E-01$)	0.71 (sd $1.03E-01$)
K-NN (N=20)	0.53 (sd $6.67E-02$)	0.55 (sd $2.07E-01$)	0.52 (sd $1.04E-01$)	0.76 (sd $1.10E-01$)
K-NN (N=50)	0.50 (sd $0.00E+00$)	0.44 (sd $5.55E-17$)	0.47 (sd $0.00E+00$)	0.75 (sd $1.43E-01$)
Gradient Boosting (exponential)	0.62 (sd $1.34E-01$)	0.61 (sd $1.48E-01$)	0.61 (sd $1.37E-01$)	0.86 (sd $8.87E-02$)
Gradient Boosting (deviance)	0.59 (sd $1.07E-01$)	0.58 (sd $1.30E-01$)	0.58 (sd $1.16E-01$)	0.83 (sd $1.09E-01$)
Balanced RF	0.81 (sd $1.07E-01$)	0.65 (sd $6.97E-02$)	0.64 (sd $1.08E-01$)	0.86 (sd $1.10E-01$)
Balanced ADABOOST	0.75 (sd $9.63E-02$)	0.69 (sd $9.33E-02$)	0.70 (sd $9.43E-02$)	0.86 (sd $6.77E-02$)

Table 43: Classification metrics for all classifiers evaluated using global network properties in $D_{[1000,+\infty]}$. Mainstream training and test data include only Right sources.

Evaluation Metrics for Classifiers in D_{all}

**Classifier**	Recall	Precision	F1-Score	AUROC
SVC Linear	0.50 (sd $0.00E+00$)	0.42 (sd $0.00E+00$)	0.46 (sd $5.55E-17$)	0.71 (sd $4.59E-02$)
SVC RBF	0.58 (sd $2.62E-02$)	0.80 (sd $4.37E-02$)	0.60 (sd $3.74E-02$)	0.66 (sd $4.89E-02$)
Logistic Regression	0.53 (sd $1.90E-02$)	0.74 (sd $9.15E-02$)	0.52 (sd $3.43E-02$)	0.78 (sd $2.60E-02$)
Random Forest	0.60 (sd $3.90E-02$)	0.68 (sd $3.90E-02$)	0.61 (sd $3.96E-02$)	0.77 (sd $1.98E-02$)
K-NN (N=5)	0.61 (sd $3.73E-02$)	0.67 (sd $3.06E-02$)	0.62 (sd $3.65E-02$)	0.72 (sd $2.43E-02$)
K-NN (N=10)	0.62 (sd $4.01E-02$)	0.70 (sd $2.72E-02$)	0.63 (sd $3.89E-02$)	0.76 (sd $2.69E-02$)
K-NN (N=20)	0.60 (sd $4.21E-02$)	0.76 (sd $5.20E-02$)	0.62 (sd $4.93E-02$)	0.78 (sd $2.38E-02$)
K-NN (N=50)	0.58 (sd $3.08E-02$)	0.79 (sd $5.20E-02$)	0.60 (sd $4.34E-02$)	0.80 (sd $2.04E-02$)
Gradient Boosting (exponential)	0.59 (sd $4.79E-02$)	0.73 (sd $9.22E-02$)	0.60 (sd $5.34E-02$)	0.80 (sd $1.81E-02$)
Gradient Boosting (deviance)	0.58 (sd $4.22E-02$)	0.73 (sd $7.75E-02$)	0.59 (sd $5.47E-02$)	0.80 (sd $2.48E-02$)
Balanced RF	0.71 (sd $2.02E-02$)	0.62 (sd $1.22E-02$)	0.60 (sd $3.48E-02$)	0.79 (sd $1.67E-02$)
Balanced ADABOOST	0.71 (sd $3.00E-02$)	0.62 (sd $1.94E-02$)	0.59 (sd $5.79E-02$)	0.78 (sd $4.22E-02$)

Table 44: Classification metrics for all classifiers evaluated using global network properties in D_{all}. Mainstream training and test data include only Centre sources.

Evaluation Metrics for Classifiers in $D_{[0,100]}$

**Classifier**	Recall	Precision	F1-Score	AUROC
SVC Linear	0.50 (sd $0.00E+00$)	0.40 (sd $5.55E-17$)	0.44 (sd $0.00E+00$)	0.61 (sd $8.87E-02$)
SVC RBF	0.61 (sd $9.73E-03$)	0.83 (sd $3.56E-02$)	0.63 (sd $1.40E-02$)	0.62 (sd $2.48E-02$)
Logistic Regression	0.56 (sd $1.62E-02$)	0.83 (sd $4.11E-02$)	0.55 (sd $2.72E-02$)	0.75 (sd $1.80E-02$)
Random Forest	0.62 (sd $2.06E-02$)	0.69 (sd $3.65E-02$)	0.64 (sd $2.47E-02$)	0.75 (sd $2.44E-02$)
K-NN (N=5)	0.62 (sd $1.82E-02$)	0.69 (sd $2.49E-02$)	0.64 (sd $2.10E-02$)	0.71 (sd $2.62E-02$)
K-NN (N=10)	0.63 (sd $2.29E-02$)	0.72 (sd $4.22E-02$)	0.65 (sd $2.77E-02$)	0.73 (sd $2.74E-02$)
K-NN (N=20)	0.61 (sd $1.48E-02$)	0.75 (sd $4.43E-02$)	0.63 (sd $1.99E-02$)	0.75 (sd $1.87E-02$)
K-NN (N=50)	0.60 (sd $1.40E-02$)	0.80 (sd $5.58E-02$)	0.63 (sd $2.00E-02$)	0.78 (sd $2.44E-02$)
Gradient Boosting (exponential)	0.62 (sd $1.25E-02$)	0.79 (sd $3.41E-02$)	0.64 (sd $1.69E-02$)	0.77 (sd $2.46E-02$)
Gradient Boosting (deviance)	0.62 (sd $1.09E-02$)	0.78 (sd $2.54E-02$)	0.64 (sd $1.42E-02$)	0.77 (sd $2.35E-02$)
Balanced RF	0.69 (sd $2.54E-02$)	0.63 (sd $1.64E-02$)	0.63 (sd $1.79E-02$)	0.77 (sd $2.44E-02$)
Balanced ADABOOST	0.69 (sd $2.81E-02$)	0.62 (sd $1.91E-02$)	0.61 (sd $2.42E-02$)	0.75 (sd $2.67E-02$)

Table 45: Classification metrics for all classifiers evaluated using global network properties in $D_{[0,100]}$. Mainstream training and test data include only Centre sources.

Evaluation Metrics for Classifiers in $D_{[100,1000]}$

**Classifier**	Recall	Precision	F1-Score	AUROC
SVC Linear	0.50 (sd $0.00E+00$)	0.44 (sd $0.00E+00$)	0.47 (sd $5.55E-17$)	0.84 (sd $2.80E-02$)
SVC RBF	0.58 (sd $1.52E-02$)	0.90 (sd $5.15E-02$)	0.60 (sd $2.26E-02$)	0.70 (sd $4.20E-02$)
Logistic Regression	0.66 (sd $5.94E-02$)	0.77 (sd $9.60E-02$)	0.68 (sd $5.18E-02$)	0.85 (sd $2.92E-02$)
Random Forest	0.64 (sd $2.94E-02$)	0.77 (sd $5.69E-02$)	0.67 (sd $3.01E-02$)	0.83 (sd $2.46E-02$)
K-NN (N=5)	0.62 (sd $2.99E-02$)	0.72 (sd $3.22E-02$)	0.64 (sd $3.16E-02$)	0.79 (sd $2.52E-02$)
K-NN (N=10)	0.62 (sd $2.55E-02$)	0.74 (sd $4.37E-02$)	0.65 (sd $3.11E-02$)	0.81 (sd $2.87E-02$)
K-NN (N=20)	0.62 (sd $2.12E-02$)	0.78 (sd $5.86E-02$)	0.65 (sd $2.86E-02$)	0.83 (sd $2.22E-02$)
K-NN (N=50)	0.61 (sd $1.55E-02$)	0.84 (sd $4.65E-02$)	0.65 (sd $2.02E-02$)	0.84 (sd $2.43E-02$)
Gradient Boosting (exponential)	0.62 (sd $1.99E-02$)	0.79 (sd $5.86E-02$)	0.65 (sd $2.37E-02$)	0.85 (sd $2.18E-02$)
Gradient Boosting (deviance)	0.63 (sd $1.96E-02$)	0.78 (sd $6.38E-02$)	0.66 (sd $2.44E-02$)	0.84 (sd $2.06E-02$)
Balanced RF	0.76 (sd $2.75E-02$)	0.63 (sd $1.21E-02$)	0.62 (sd $1.61E-02$)	0.85 (sd $1.90E-02$)
Balanced ADABOOST	0.75 (sd $3.54E-02$)	0.63 (sd $1.77E-02$)	0.64 (sd $2.28E-02$)	0.84 (sd $2.54E-02$)

Table 46: Classification metrics for all classifiers evaluated using global network properties in $D_{[100,1000]}$. Mainstream training and test data include only Centre sources.

Evaluation Metrics for Classifiers in $D_{[1000,+\infty]}$				
**Classifier**	Recall	Precision	F1-Score	AUROC
SVC Linear	0.50 (sd $0.00E+00$)	0.46 (sd $0.00E+00$)	0.48 (sd $5.55E-17$)	0.87 (sd $1.64E-01$)
SVC RBF	0.50 (sd $6.25E-03$)	0.46 (sd $4.62E-04$)	0.48 (sd $3.18E-03$)	0.81 (sd $1.74E-01$)
Logistic Regression	0.61 (sd $1.71E-01$)	0.62 (sd $2.12E-01$)	0.61 (sd $1.84E-01$)	0.85 (sd $1.90E-01$)
Random Forest	0.69 (sd $1.48E-01$)	0.73 (sd $2.03E-01$)	0.70 (sd $1.64E-01$)	0.84 (sd $1.61E-01$)
K-NN (N=5)	0.59 (sd $1.20E-01$)	0.64 (sd $2.32E-01$)	0.60 (sd $1.56E-01$)	0.77 (sd $1.87E-01$)
K-NN (N=10)	0.50 (sd $6.25E-03$)	0.46 (sd $4.62E-04$)	0.48 (sd $3.18E-03$)	0.82 (sd $1.49E-01$)
K-NN (N=20)	0.50 (sd $0.00E+00$)	0.46 (sd $0.00E+00$)	0.48 (sd $5.55E-17$)	0.80 (sd $1.73E-01$)
K-NN (N=50)	0.50 (sd $0.00E+00$)	0.46 (sd $0.00E+00$)	0.48 (sd $5.55E-17$)	0.80 (sd $2.09E-01$)
Gradient Boosting (exponential)	0.66 (sd $1.51E-01$)	0.65 (sd $1.74E-01$)	0.65 (sd $1.49E-01$)	0.85 (sd $1.34E-01$)
Gradient Boosting (deviance)	0.65 (sd $1.44E-01$)	0.60 (sd $1.22E-01$)	0.62 (sd $1.23E-01$)	0.82 (sd $1.66E-01$)
Balanced RF	0.78 (sd $1.07E-01$)	0.64 (sd $1.25E-01$)	0.61 (sd $1.17E-01$)	0.85 (sd $1.68E-01$)
Balanced ADABOOST	0.72 (sd $1.66E-01$)	0.64 (sd $1.43E-01$)	0.65 (sd $1.21E-01$)	0.83 (sd $1.42E-01$)

Table 47: Classification metrics for all classifiers evaluated using global network properties in $D_{[1000,+\infty]}$. Mainstream training and test data include only Centre sources.

Evaluation Metrics for Classifiers in D_{all}				
**Classifier**	Recall	Precision	F1-Score	AUROC
Balanced RF	0.69 (sd $3.02E-02$)	0.72 (sd $3.02E-02$)	0.69 (sd $3.35E-02$)	0.76 (sd $3.04E-02$)
Balanced ADABOOST	0.67 (sd $3.95E-02$)	0.70 (sd $2.46E-02$)	0.67 (sd $4.66E-02$)	0.75 (sd $3.66E-02$)
SVC Linear	0.59 (sd $4.74E-02$)	0.77 (sd $1.12E-02$)	0.52 (sd $7.95E-02$)	0.74 (sd $2.61E-02$)
SVC RBF	0.58 (sd $2.94E-02$)	0.77 (sd $1.41E-02$)	0.52 (sd $4.98E-02$)	0.70 (sd $2.88E-02$)
Logistic Regression	0.58 (sd $2.02E-02$)	0.77 (sd $1.13E-02$)	0.51 (sd $3.52E-02$)	0.77 (sd $2.14E-02$)
Random Forest	0.61 (sd $4.14E-02$)	0.76 (sd $2.16E-02$)	0.56 (sd $6.44E-02$)	0.74 (sd $2.98E-02$)
K-NN (N=5)	0.62 (sd $3.51E-02$)	0.74 (sd $2.46E-02$)	0.58 (sd $5.33E-02$)	0.71 (sd $3.06E-02$)
K-NN (N=10)	0.61 (sd $3.38E-02$)	0.77 (sd $1.44E-02$)	0.55 (sd $5.55E-02$)	0.75 (sd $2.36E-02$)
K-NN (N=20)	0.61 (sd $3.66E-02$)	0.77 (sd $1.25E-02$)	0.56 (sd $5.93E-02$)	0.77 (sd $2.03E-02$)
K-NN (N=50)	0.60 (sd $3.06E-02$)	0.76 (sd $1.24E-02$)	0.55 (sd $5.03E-02$)	0.79 (sd $1.24E-02$)
Gradient Boosting (exponential)	0.60 (sd $4.33E-02$)	0.77 (sd $9.61E-03$)	0.54 (sd $7.15E-02$)	0.75 (sd $3.90E-02$)
Gradient Boosting (deviance)	0.60 (sd $4.18E-02$)	0.76 (sd $1.56E-02$)	0.55 (sd $6.69E-02$)	0.75 (sd $4.17E-02$)

Table 48: Classification metrics for classifiers evaluated using global network properties in D_{all}. Training on left-biased Mainstream and Misleading sources only and testing on all sources regardless of bias.

Evaluation Metrics for Classifiers in $D_{[0,100]}$				
**Classifier**	Recall	Precision	F1-Score	AUROC
Balanced RF	0.70 (sd $1.79E-02$)	0.69 (sd $1.71E-02$)	0.69 (sd $1.74E-02$)	0.78 (sd $1.38E-02$)
Balanced ADABOOST	0.67 (sd $5.34E-03$)	0.67 (sd $6.12E-03$)	0.67 (sd $5.69E-03$)	0.76 (sd $1.11E-02$)
SVC Linear	0.59 (sd $6.94E-03$)	0.78 (sd $9.89E-03$)	0.56 (sd $1.06E-02$)	0.64 (sd $4.32E-02$)
SVC RBF	0.52 (sd $9.81E-03$)	0.72 (sd $4.44E-02$)	0.42 (sd $1.99E-02$)	0.68 (sd $2.30E-02$)
Logistic Regression	0.56 (sd $1.07E-02$)	0.76 (sd $2.60E-02$)	0.50 (sd $1.78E-02$)	0.74 (sd $1.80E-02$)
Random Forest	0.61 (sd $1.30E-02$)	0.76 (sd $2.39E-02$)	0.59 (sd $1.85E-02$)	0.77 (sd $1.49E-02$)
K-NN (N=5)	0.61 (sd $1.32E-02$)	0.74 (sd $1.21E-02$)	0.59 (sd $2.00E-02$)	0.73 (sd $1.16E-02$)
K-NN (N=10)	0.59 (sd $1.10E-02$)	0.77 (sd $1.72E-02$)	0.56 (sd $1.75E-02$)	0.74 (sd $1.30E-02$)
K-NN (N=20)	0.60 (sd $9.89E-03$)	0.78 (sd $1.59E-02$)	0.56 (sd $1.51E-02$)	0.76 (sd $1.58E-02$)
K-NN (N=50)	0.59 (sd $8.52E-03$)	0.79 (sd $1.08E-02$)	0.55 (sd $1.38E-02$)	0.76 (sd $1.46E-02$)
Gradient Boosting (exponential)	0.59 (sd $7.79E-03$)	0.78 (sd $1.82E-02$)	0.56 (sd $1.15E-02$)	0.78 (sd $1.58E-02$)
Gradient Boosting (deviance)	0.60 (sd $1.04E-02$)	0.78 (sd $1.70E-02$)	0.57 (sd $1.49E-02$)	0.78 (sd $1.40E-02$)

Table 49: Classification metrics for classifiers evaluated using global network properties in $D_{[0,100]}$. Training on left-biased Mainstream and Misleading sources only and testing on all sources regardless of bias.

Classifier	Recall	Precision	F1-Score	AUROC
	**Evaluation Metrics for Classifiers in $D_{[100,1000]}$**			
Balanced RF	0.75 (sd 3.89$E-02$)	0.76 (sd 3.24$E-02$)	0.75 (sd 4.33$E-02$)	0.84 (sd 3.10$E-02$)
Balanced ADABOOST	0.72 (sd 2.36$E-02$)	0.73 (sd 1.86$E-02$)	0.71 (sd 2.72$E-02$)	0.81 (sd 3.13$E-02$)
SVC Linear	0.68 (sd 5.79$E-02$)	0.75 (sd 1.64$E-02$)	0.64 (sd 9.06$E-02$)	0.84 (sd 1.14$E-02$)
SVC RBF	0.65 (sd 3.81$E-02$)	0.76 (sd 1.05$E-02$)	0.59 (sd 5.97$E-02$)	0.75 (sd 1.82$E-02$)
Logistic Regression	0.66 (sd 3.19$E-02$)	0.77 (sd 1.33$E-02$)	0.61 (sd 4.67$E-02$)	0.85 (sd 1.86$E-02$)
Random Forest	0.69 (sd 5.14$E-02$)	0.77 (sd 2.76$E-02$)	0.65 (sd 7.42$E-02$)	0.81 (sd 4.31$E-02$)
K-NN (N=5)	0.69 (sd 4.46$E-02$)	0.76 (sd 2.54$E-02$)	0.66 (sd 6.11$E-02$)	0.80 (sd 2.68$E-02$)
K-NN (N=10)	0.67 (sd 4.12$E-02$)	0.77 (sd 1.43$E-02$)	0.62 (sd 6.31$E-02$)	0.82 (sd 2.89$E-02$)
K-NN (N=20)	0.66 (sd 4.33$E-02$)	0.76 (sd 1.70$E-02$)	0.60 (sd 6.65$E-02$)	0.83 (sd 2.14$E-02$)
K-NN (N=50)	0.64 (sd 4.16$E-02$)	0.76 (sd 1.11$E-02$)	0.57 (sd 6.82$E-02$)	0.84 (sd 1.74$E-02$)
Gradient Boosting (exponential)	0.68 (sd 5.11$E-02$)	0.77 (sd 2.24$E-02$)	0.64 (sd 7.52$E-02$)	0.82 (sd 4.09$E-02$)
Gradient Boosting (deviance)	0.68 (sd 5.00$E-02$)	0.77 (sd 2.01$E-02$)	0.64 (sd 7.39$E-02$)	0.82 (sd 4.17$E-02$)

Table 50: Classification metrics for classifiers evaluated using global network properties in $D_{[100,1000]}$. Training on left-biased Mainstream and Misleading sources only and testing on all sources regardless of bias.

Classifier	Recall	Precision	F1-Score	AUROC
	**Evaluation Metrics for Classifiers in $D_{[1000,+\infty]}$**			
Balanced RF	0.79 (sd 5.84$E-02$)	0.79 (sd 6.02$E-02$)	0.78 (sd 5.80$E-02$)	0.90 (sd 3.88$E-02$)
Balanced ADABOOST	0.79 (sd 4.17$E-02$)	0.79 (sd 4.33$E-02$)	0.78 (sd 4.21$E-02$)	0.87 (sd 3.49$E-02$)
SVC Linear	0.54 (sd 1.46$E-02$)	0.74 (sd 3.96$E-03$)	0.39 (sd 2.86$E-02$)	0.93 (sd 3.38$E-02$)
SVC RBF	0.55 (sd 2.78$E-02$)	0.68 (sd 1.56$E-01$)	0.42 (sd 4.62$E-02$)	0.75 (sd 5.65$E-02$)
Logistic Regression	0.68 (sd 5.04$E-02$)	0.77 (sd 3.92$E-02$)	0.63 (sd 6.61$E-02$)	0.91 (sd 3.81$E-02$)
Random Forest	0.72 (sd 4.84$E-02$)	0.78 (sd 3.59$E-02$)	0.69 (sd 5.81$E-02$)	0.90 (sd 4.62$E-02$)
K-NN (N=5)	0.67 (sd 5.12$E-02$)	0.74 (sd 5.01$E-02$)	0.63 (sd 6.20$E-02$)	0.81 (sd 4.45$E-02$)
K-NN (N=10)	0.59 (sd 4.47$E-02$)	0.73 (sd 5.33$E-02$)	0.49 (sd 6.80$E-02$)	0.81 (sd 5.01$E-02$)
K-NN (N=20)	0.55 (sd 1.91$E-02$)	0.74 (sd 5.29$E-03$)	0.41 (sd 3.66$E-02$)	0.79 (sd 5.97$E-02$)
K-NN (N=50)	0.51 (sd 9.55$E-03$)	0.38 (sd 2.32$E-01$)	0.33 (sd 2.06$E-02$)	0.76 (sd 7.36$E-02$)
Gradient Boosting (exponential)	0.75 (sd 5.63$E-02$)	0.78 (sd 4.43$E-02$)	0.74 (sd 6.75$E-02$)	0.87 (sd 5.12$E-02$)
Gradient Boosting (deviance)	0.75 (sd 5.20$E-02$)	0.77 (sd 4.73$E-02$)	0.74 (sd 5.88$E-02$)	0.87 (sd 5.27$E-02$)

Table 51: Classification metrics for classifiers evaluated using global network properties in $D_{[1000,+\infty]}$. Training on left-biased Mainstream and Misleading sources only and testing on all sources regardless of bias.

Classifier	Recall	Precision	F1-Score	AUROC
	**Evaluation Metrics for Classifiers in D_{all}**			
Balanced RF	0.70 (sd 2.05$E-02$)	0.70 (sd 2.26$E-02$)	0.70 (sd 2.21$E-02$)	0.78 (sd 1.79$E-02$)
Balanced ADABOOST	0.70 (sd 2.52$E-02$)	0.70 (sd 2.32$E-02$)	0.69 (sd 2.69$E-02$)	0.77 (sd 2.30$E-02$)
SVC Linear	0.50 (sd 0.00$E+00$)	0.23 (sd 2.78$E-17$)	0.31 (sd 5.55$E-17$)	0.76 (sd 2.64$E-02$)
SVC RBF	0.53 (sd 2.41$E-02$)	0.72 (sd 2.27$E-02$)	0.38 (sd 4.93$E-02$)	0.60 (sd 6.95$E-02$)
Logistic Regression	0.52 (sd 1.24$E-02$)	0.71 (sd 1.75$E-02$)	0.36 (sd 2.75$E-02$)	0.77 (sd 1.18$E-02$)
Random Forest	0.58 (sd 4.06$E-02$)	0.66 (sd 2.37$E-02$)	0.50 (sd 7.62$E-02$)	0.76 (sd 1.73$E-02$)
K-NN (N=5)	0.59 (sd 3.85$E-02$)	0.65 (sd 3.13$E-02$)	0.52 (sd 5.95$E-02$)	0.71 (sd 2.91$E-02$)
K-NN (N=10)	0.59 (sd 4.19$E-02$)	0.66 (sd 3.56$E-02$)	0.51 (sd 6.80$E-02$)	0.74 (sd 2.56$E-02$)
K-NN (N=20)	0.56 (sd 4.30$E-02$)	0.69 (sd 2.29$E-02$)	0.45 (sd 8.33$E-02$)	0.77 (sd 2.01$E-02$)
K-NN (N=50)	0.55 (sd 3.86$E-02$)	0.70 (sd 1.88$E-02$)	0.42 (sd 7.87$E-02$)	0.78 (sd 1.44$E-02$)
Gradient Boosting (exponential)	0.55 (sd 5.00$E-02$)	0.70 (sd 2.47$E-02$)	0.43 (sd 1.05$E-01$)	0.78 (sd 1.29$E-02$)
Gradient Boosting (deviance)	0.56 (sd 5.15$E-02$)	0.71 (sd 2.28$E-02$)	0.43 (sd 1.06$E-01$)	0.78 (sd 1.31$E-02$)

Table 52: Classification metrics for classifiers evaluated using global network properties in D_{all}. Training on right-biased Mainstream and Misleading sources only and testing on all sources regardless of bias.

Evaluation Metrics for Classifiers in $D_{[0,100]}$

Classifier	Recall	Precision	F1-Score	AUROC
Balanced RF	0.69 (sd $1.29E-02$)	0.68 (sd $1.22E-02$)	0.68 (sd $1.21E-02$)	0.78 (sd $1.52E-02$)
Balanced ADABOOST	0.68 (sd $1.98E-02$)	0.67 (sd $1.89E-02$)	0.67 (sd $1.92E-02$)	0.75 (sd $1.99E-02$)
SVC Linear	0.50 (sd $0.00E+00$)	0.19 (sd $0.00E+00$)	0.28 (sd $5.55E-17$)	0.70 (sd $2.08E-02$)
SVC RBF	0.60 (sd $1.01E-02$)	0.69 (sd $1.36E-02$)	0.49 (sd $1.46E-02$)	0.67 (sd $1.39E-02$)
Logistic Regression	0.59 (sd $8.37E-03$)	0.68 (sd $8.85E-03$)	0.46 (sd $1.63E-02$)	0.74 (sd $1.75E-02$)
Random Forest	0.64 (sd $1.59E-02$)	0.67 (sd $1.76E-02$)	0.57 (sd $1.89E-02$)	0.76 (sd $1.69E-02$)
K-NN (N=5)	0.64 (sd $1.30E-02$)	0.67 (sd $1.47E-02$)	0.57 (sd $1.40E-02$)	0.74 (sd $1.70E-02$)
K-NN (N=10)	0.65 (sd $1.11E-02$)	0.67 (sd $1.19E-02$)	0.59 (sd $1.20E-02$)	0.76 (sd $1.12E-02$)
K-NN (N=20)	0.63 (sd $1.13E-02$)	0.67 (sd $1.50E-02$)	0.56 (sd $1.14E-02$)	0.76 (sd $1.51E-02$)
K-NN (N=50)	0.61 (sd $8.07E-03$)	0.69 (sd $1.07E-02$)	0.51 (sd $1.12E-02$)	0.76 (sd $1.53E-02$)
Gradient Boosting (exponential)	0.61 (sd $1.12E-02$)	0.69 (sd $1.30E-02$)	0.50 (sd $1.58E-02$)	0.76 (sd $1.82E-02$)
Gradient Boosting (deviance)	0.61 (sd $1.27E-02$)	0.69 (sd $1.44E-02$)	0.50 (sd $1.79E-02$)	0.76 (sd $1.66E-02$)

Table 53: Classification metrics for classifiers evaluated using global network properties in $D_{[0,100]}$. Training on right-biased Mainstream and Misleading sources only and testing on all sources regardless of bias.

Evaluation Metrics for Classifiers in $D_{[100,1000]}$

Classifier	Recall	Precision	F1-Score	AUROC
Balanced RF	0.76 (sd $1.17E-02$)	0.76 (sd $1.19E-02$)	0.76 (sd $1.18E-02$)	0.85 (sd $1.16E-02$)
Balanced ADABOOST	0.73 (sd $2.44E-02$)	0.74 (sd $1.96E-02$)	0.73 (sd $2.56E-02$)	0.83 (sd $1.34E-02$)
SVC Linear	0.52 (sd $6.15E-03$)	0.74 (sd $4.43E-02$)	0.38 (sd $1.19E-02$)	0.85 (sd $1.72E-02$)
SVC RBF	0.57 (sd $8.55E-03$)	0.77 (sd $1.31E-02$)	0.49 (sd $1.50E-02$)	0.71 (sd $3.12E-02$)
Logistic Regression	0.59 (sd $4.77E-02$)	0.76 (sd $2.35E-02$)	0.52 (sd $8.04E-02$)	0.84 (sd $1.07E-02$)
Random Forest	0.63 (sd $1.94E-02$)	0.76 (sd $1.61E-02$)	0.59 (sd $2.96E-02$)	0.84 (sd $1.65E-02$)
K-NN (N=5)	0.64 (sd $1.80E-02$)	0.74 (sd $2.00E-02$)	0.61 (sd $2.46E-02$)	0.79 (sd $1.30E-02$)
K-NN (N=10)	0.64 (sd $9.29E-03$)	0.76 (sd $1.59E-02$)	0.61 (sd $1.18E-02$)	0.82 (sd $1.58E-02$)
K-NN (N=20)	0.62 (sd $6.56E-03$)	0.76 (sd $1.01E-02$)	0.57 (sd $1.02E-02$)	0.84 (sd $1.09E-02$)
K-NN (N=50)	0.61 (sd $1.03E-02$)	0.76 (sd $8.99E-03$)	0.55 (sd $1.74E-02$)	0.84 (sd $1.17E-02$)
Gradient Boosting (exponential)	0.61 (sd $1.80E-02$)	0.76 (sd $1.34E-02$)	0.55 (sd $2.86E-02$)	0.84 (sd $1.00E-02$)
Gradient Boosting (deviance)	0.61 (sd $1.55E-02$)	0.75 (sd $1.71E-02$)	0.55 (sd $2.44E-02$)	0.84 (sd $1.04E-02$)

Table 54: Classification metrics for classifiers evaluated using global network properties in $D_{[100,1000]}$. Training on right-biased Mainstream and Misleading sources only and testing on all sources regardless of bias.

Evaluation Metrics for Classifiers in $D_{[1000,+\infty]}$

Classifier	Recall	Precision	F1-Score	AUROC
Balanced RF	0.77 (sd $1.15E-01$)	0.80 (sd $1.03E-01$)	0.76 (sd $1.34E-01$)	0.89 (sd $6.53E-02$)
Balanced ADABOOST	0.72 (sd $9.75E-02$)	0.82 (sd $5.17E-02$)	0.71 (sd $1.26E-01$)	0.87 (sd $8.51E-02$)
SVC Linear	0.50 (sd $0.00E+00$)	0.27 (sd $5.55E-17$)	0.35 (sd $0.00E+00$)	0.92 (sd $3.29E-02$)
SVC RBF	0.53 (sd $3.17E-02$)	0.58 (sd $2.52E-01$)	0.42 (sd $6.14E-02$)	0.80 (sd $7.39E-02$)
Logistic Regression	0.55 (sd $2.45E-02$)	0.73 (sd $1.54E-01$)	0.44 (sd $4.72E-02$)	0.91 (sd $3.65E-02$)
Random Forest	0.62 (sd $8.86E-02$)	0.76 (sd $1.64E-01$)	0.56 (sd $1.40E-01$)	0.85 (sd $9.94E-02$)
K-NN (N=5)	0.59 (sd $4.64E-02$)	0.80 (sd $1.40E-02$)	0.52 (sd $7.61E-02$)	0.78 (sd $5.31E-02$)
K-NN (N=10)	0.56 (sd $2.97E-02$)	0.79 (sd $8.32E-03$)	0.47 (sd $5.34E-02$)	0.81 (sd $4.54E-02$)
K-NN (N=20)	0.52 (sd $1.50E-02$)	0.63 (sd $2.32E-01$)	0.39 (sd $3.09E-02$)	0.82 (sd $6.35E-02$)
K-NN (N=50)	0.50 (sd $0.00E+00$)	0.27 (sd $5.55E-17$)	0.35 (sd $0.00E+00$)	0.85 (sd $5.01E-02$)
Gradient Boosting (exponential)	0.62 (sd $8.88E-02$)	0.70 (sd $2.14E-01$)	0.55 (sd $1.41E-01$)	0.84 (sd $1.14E-01$)
Gradient Boosting (deviance)	0.63 (sd $1.01E-01$)	0.70 (sd $2.16E-01$)	0.58 (sd $1.57E-01$)	0.83 (sd $1.20E-01$)

Table 55: Classification metrics for classifiers evaluated using global network properties in $D_{[1000,+\infty]}$. Training on right-biased Mainstream and Misleading sources only and testing on all sources regardless of bias.

6 Box-plots for the distribution of features taking into account bias of sources

In this section we provide box-plots in all subsets for the empirical distributions of all features per different bias of sources, similar to Section 3.2.

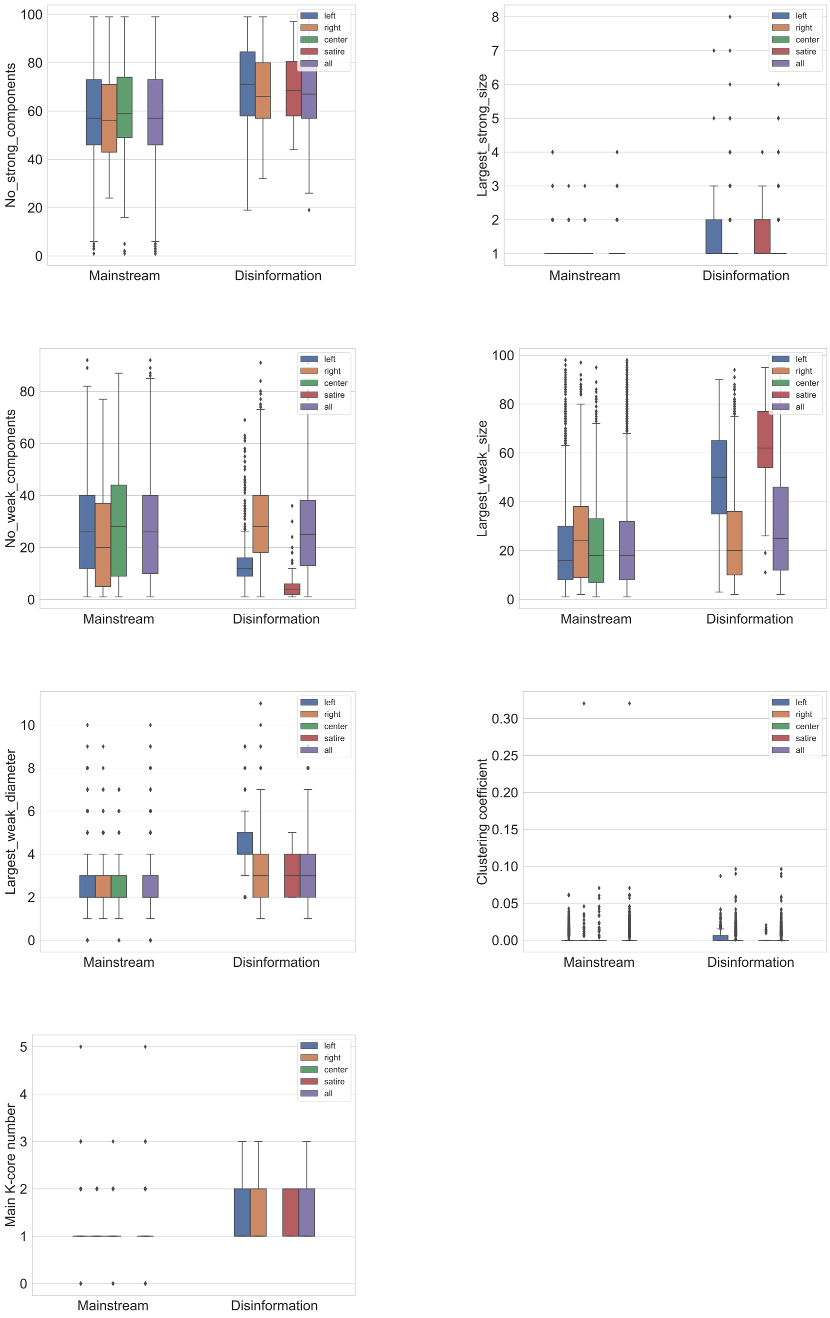

Figure 11: Box plots for all global network properties in $D_{[0,100)}$

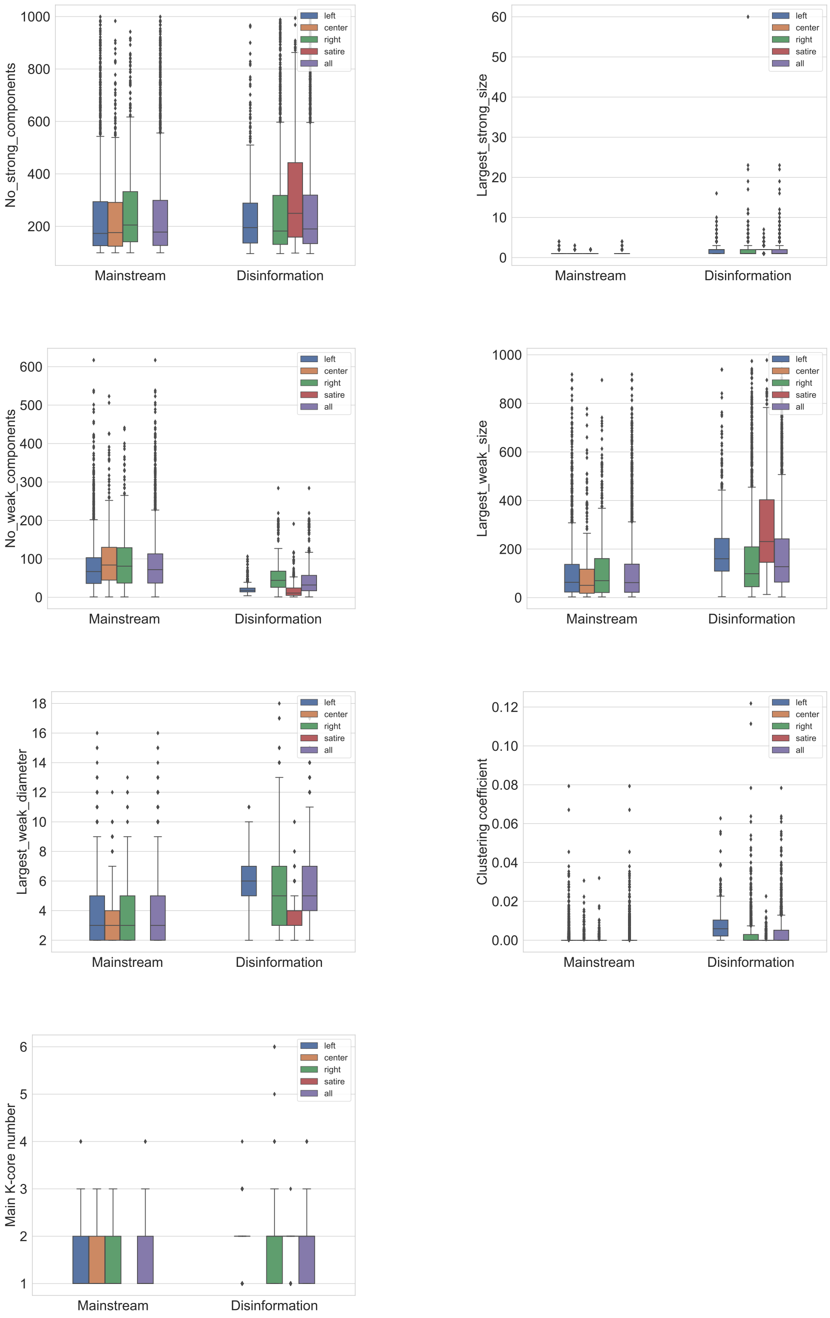

Figure 12: Box plots for all global network properties in $D_{[100,1000)}$

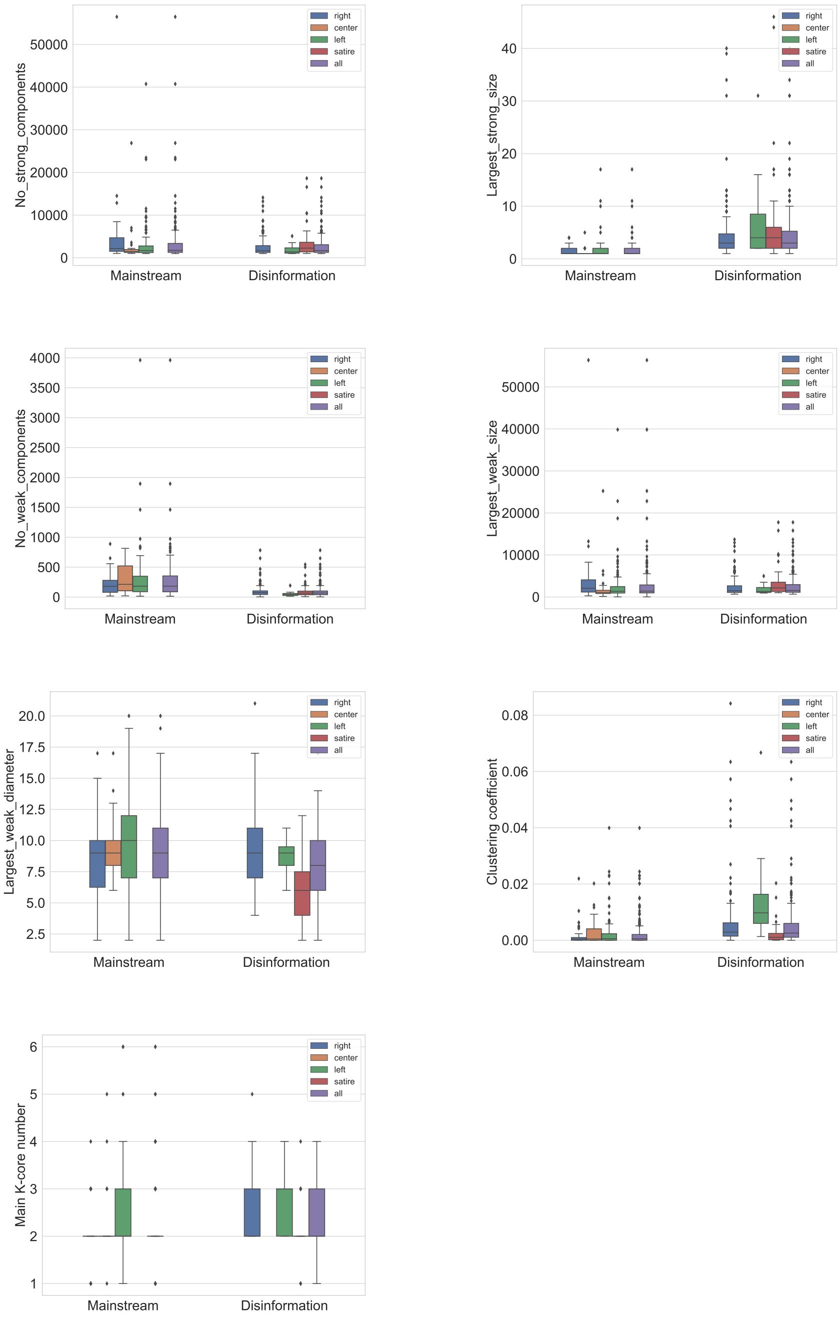

Figure 13: Box plots for all global network properties in $D_{[1000,+\infty)}$

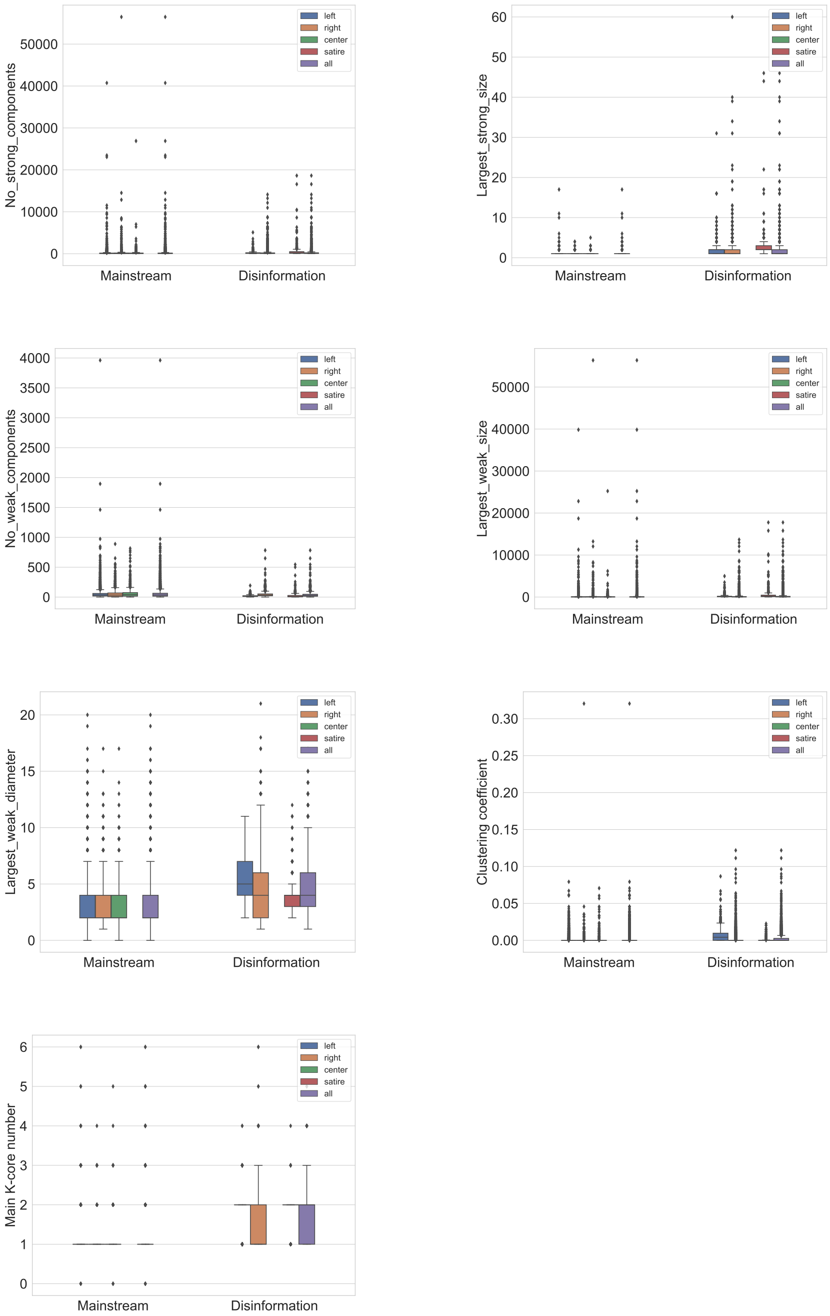

Figure 14: Box plots for all global network properties in D_{all}

7 Networks Plots

In this section we provide some example plots for networks belonging to both news domains. We used two different strategies to identify most appropriate individuals to plot:

1. *nearest* individual: the network with the smallest Euclidean distance from all other individuals in the same domain, using the vectors of global network properties;

2. *farthest* individual: the network with the highest Euclidean distance from all other individuals in the other domain, using the vectors of global network properties.

Plots were obtained using Gephi [2] and the Force Atlas 2 visualization algorithm with parameters: Stronger Gravity = ON, Approximate Repulsion = ON, Prevent Overlap = ON, Scaling = 100. We also adjust node sizes according to their degree.

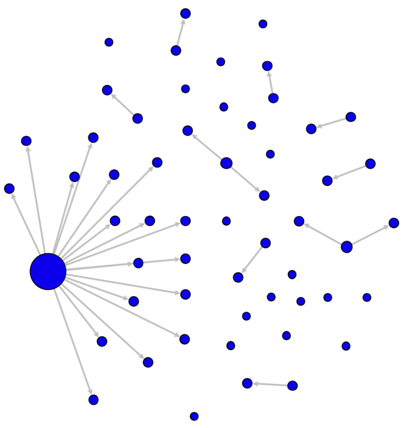

a) Mainstream diffusion network

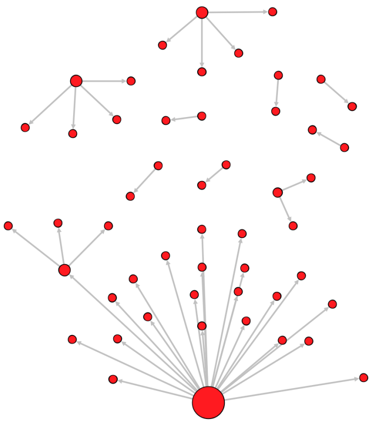

b) Misleading diffusion network

Figure 15: (bottom) The *nearest* diffusion networks in both news domains belonging to $D_{[0,100)}$. The misleading network has a larger size and diameter of the largest weakly connected component.

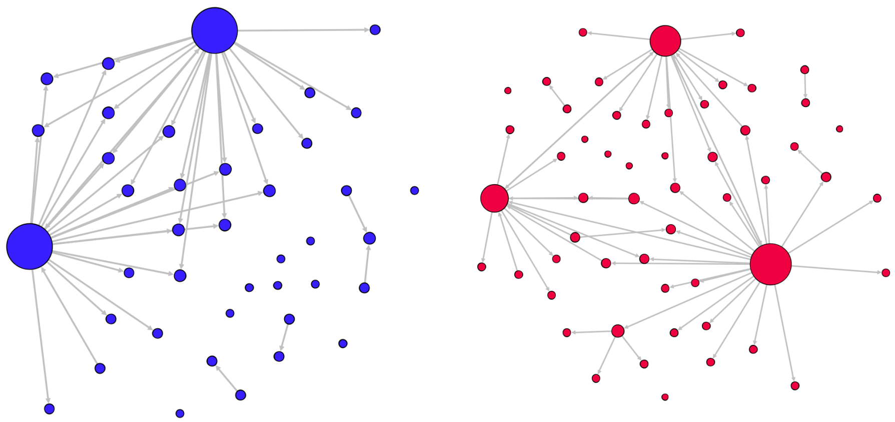

a) Mainstream diffusion network b) Misleading diffusion network

Figure 16: (bottom) The *farthest* diffusion networks in both news domains belonging to $D_{[0,100)}$. The misleading network has a larger size and diameter of the largest weakly connected component.

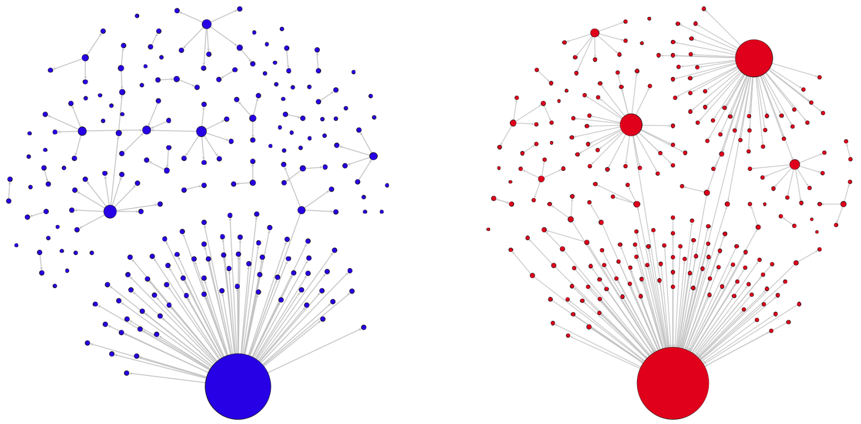

a) Mainstream diffusion network b) Misleading diffusion network

Figure 17: (bottom) The *farthest* diffusion networks in both news domains belonging to $D_{[100,1000)}$. The misleading network has a larger diameter and size of the largest weakly connected component, and a smaller number of weakly connected components.

References

[1] A.-L. Barabási. *Network science*. Cambridge University Press, 2016.

[2] M. Bastian, S. Heymann, and M. Jacomy. Gephi: An open source software for exploring and manipulating networks. In *International AAAI Conference on Weblogs and Social Media*, 2009.

[3] V. Batagelj and M. Zaversnik. An o (m) algorithm for cores decomposition of networks. *arXiv preprint cs/0310049*, 2003.

[4] A. Bovet and H. A. Makse. Influence of fake news in Twitter during the 2016 US presidential election. *Nature Communications*, 10(1):7, 2019.

[5] B. Efron and T. Hastie. *Computer age statistical inference*, volume 5. Cambridge University Press, 2016.

[6] T. Fawcett. An introduction to roc analysis. *Pattern recognition letters*, 27(8):861–874, 2006.

[7] S. Goel, A. Anderson, J. Hofman, and D. J. Watts. The structural virality of online diffusion. *Management Science*, 62(1):180–196, 2015.

[8] A. Hagberg, P. Swart, and D. S Chult. Exploring network structure, dynamics, and function using networkx. Technical report, Los Alamos National Lab.(LANL), Los Alamos, NM (United States), 2008.

[9] E. Jones, T. Oliphant, P. Peterson, et al. SciPy: Open source scientific tools for Python, 2001.

[10] G. Lemaître, F. Nogueira, and C. K. Aridas. Imbalanced-learn: A python toolbox to tackle the curse of imbalanced datasets in machine learning. *Journal of Machine Learning Research*, 18(17):1–5, 2017.

[11] W. McKinney. Data structures for statistical computing in python. In S. van der Walt and J. Millman, editors, *Proceedings of the 9th Python in Science Conference*, pages 51 – 56, 2010.

[12] A. Mitchell, J. Gottfried, J. Kiley, and K. E. Matsa. Political polarization & media habits. *Pew Research Center*, 21, 2014.

[13] M. Newman. *Networks: an introduction*. Oxford University Press, 2010.

[14] F. Pedregosa, G. Varoquaux, A. Gramfort, V. Michel, B. Thirion, O. Grisel, M. Blondel, P. Prettenhofer, R. Weiss, V. Dubourg, et al. Scikit-learn: Machine learning in python. *Journal of machine learning research*, 12(Oct):2825–2830, 2011.

[15] J. Saramäki, M. Kivelä, J.-P. Onnela, K. Kaski, and J. Kertesz. Generalizations of the clustering coefficient to weighted complex networks. *Physical Review E*, 75(2):027105, 2007.

[16] C. Shao, G. L. Ciampaglia, A. Flammini, and F. Menczer. Hoaxy: A platform for tracking online misinformation. In *Proceedings of the 25th International Conference Companion on World Wide Web*, WWW '16 Companion, pages 745–750, Republic and Canton of Geneva, Switzerland, 2016. International World Wide Web Conferences Steering Committee.

[17] C. Shao, P.-M. Hui, L. Wang, X. Jiang, A. Flammini, F. Menczer, and G. L. Ciampaglia. Anatomy of an online misinformation network. *PLOS ONE*, 13(4):1–23, 04 2018.

[18] S. Van Der Walt, S. C. Colbert, and G. Varoquaux. The numpy array: a structure for efficient numerical computation. *Computing in Science & Engineering*, 13(2):22, 2011.
